# A novel highly differentially expressed gene in wheat endosperm associated with bread quality

**DOI:** 10.1038/srep10446

**Published:** 2015-05-26

**Authors:** A. Furtado, P. C. Bundock, P. M. Banks, G. Fox, X. Yin, R. J. Henry

**Affiliations:** 1 Queensland Alliance for Agriculture and Food Innovation, The University of Queensland, St Lucia, Queensland 4072, Australia; 2 Southern Cross Plant Science, Southern Cross University, Lismore, NSW-2480, Australia; 3 Queensland Alliance for Agriculture and Food Innovation, Centre for Nutrition and Food Science, The University of Queensland, Toowoomba Qld, Australia; 4 Plant Transformation Core Facility, 1-33 Agriculture Building, Division of Plant Sciences, University of Missouri, Columbia, MO 65211, USA

## Abstract

Analysis of gene expression in developing wheat seeds was used to identify a gene, wheat bread making (*wbm*), with highly differential expression (~1000 fold) in the starchy endosperm of genotypes varying in bread making quality. Several alleles differing in the 5’-upstream region (promoter) of this gene were identified, with one present only in genotypes with high levels of *wbm* expression. RNA-Seq analysis revealed low or no *wbm* expression in most genotypes but high expression (0.2-0.4% of total gene expression) in genotypes that had good bread loaf volume. The *wbm* gene is predicted to encode a mature protein of 48 amino acids (including four cysteine residues) not previously identified in association with wheat quality, possibly because of its small size and low frequency in the wheat gene pool. Genotypes with high *wbm* expression all had good bread making quality but not always good physical dough qualities. The predicted protein was sulphur rich suggesting the possibility of a contribution to bread loaf volume by supporting the crossing linking of proteins in gluten. Improved understanding of the molecular basis of differences in bread making quality may allow more rapid development of high performing genotypes with acceptable end-use properties and facilitate increased wheat production.

Wheat is a major food crop and source of energy and protein in human diets. Hence, ongoing genetic improvements in wheat productivity are critical for food security^1^,^2^,^3^,^4^. Unlike other cereals, wheat has unique and complex qualities required for breadmaking^5^. Proteins in the wheat grain have a distinct property, such that, wheat flour when mixed with water forms an elastic mass called dough. The wheat seed proteins in dough form a complex called gluten responsible for the elasticity of dough^6^. The formation of gluten is crucial for the breadmaking process and this novel characteristic of wheat is central to its widespread consumption. Many proteins influencing the physical properties of wheat dough have been characterized^6^,^7^,^8^,^9^,^10^ but the key determinants of genetic variation in bread making quality have not been identified.

Assessing a wheat genotype for bread making quality in undertaken by a baking test, and this measurement of bread quality is only possible late in the breeding process because of the need for relatively large quantities of seed to mill and bake. Key bread quality characteristics such as loaf volume are not predictable from flour or dough quality tests^11^. The tests have relatively low repeatability requiring their assessment over several seasons and environments^12^. The failure of many advanced lines to satisfy bread tests reduces the rate of genetic gain for yield in wheat breeding^13^. An understanding of the molecular basis of this trait would be a major advantage in wheat breeding.

Advances in molecular techniques^14^ provide new opportunities to identify the genetic and molecular basis of bread quality in wheat to enable more rapid progress in satisfying global food demand. In this study we have analysed the transcriptome of developing wheat seeds^15^ to identify differentially expressed genes that may explain key differences in wheat quality. We now report the characterization of a novel wheat gene with highly differential expression in the seed and explore the association of this gene with wheat quality.

## Results

### Long-SAGE to identify differentially expressed genes in wheat seeds cv Banks and Kite

**Figure 1:**
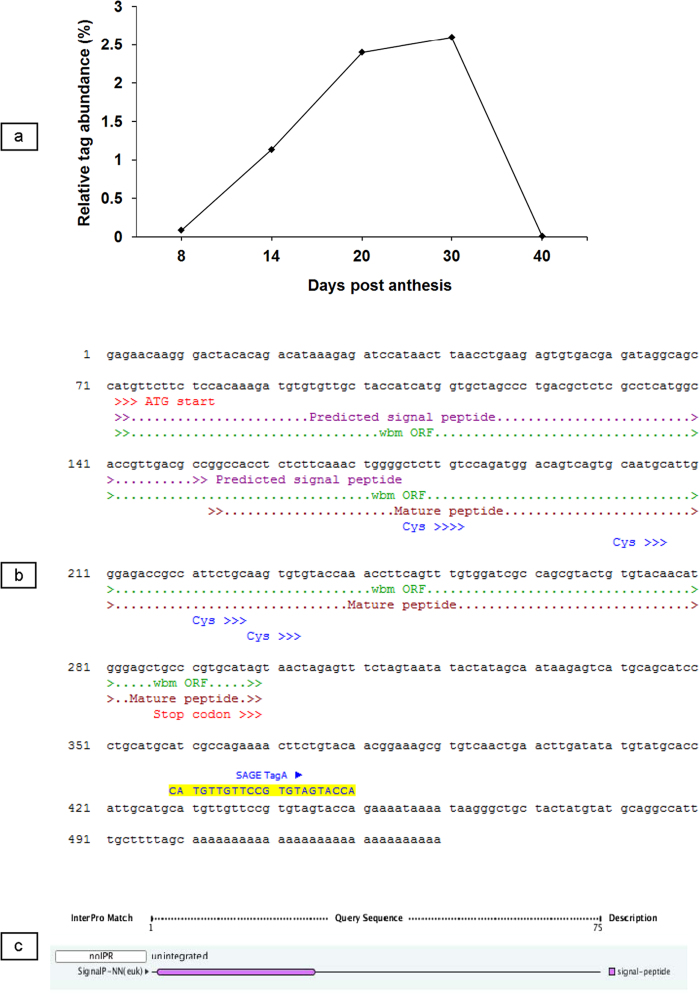
Relative abundance of transcripts and predicted sequence of a novel protein encoding gene in developing wheat seed (genotype Banks). **a** Relative abundance of transcripts during seed development based upon frequency of the tag, CATGTTGTTCCGTGTAGTACC, in LongSAGE libraries generated from developing seeds of wheat at different time points (days post anthesis); **b** Sequence identified from ESTs matching this transcript. An open reading frame with start and stop codons including the location of the tag is indicated. The contig shown is hereafter referred to as ‘*wheat bread making* gene (*wbm* gene ); **c** The *wbm* gene encodes a small protein where the open reading frame consists of a predicted signal peptide of 27 amino acid (aa) residues and a non-cytoplasmic domain which spans from 28 to 75 aa residues. Predicted signal peptide spanning the first 27aa of the contig (InterProScan, EBI, http://www.ebi.ac.uk/)

Following sorting and annotation of the first one hundred tags, those tags corresponding to the glutenin or gliadin genes (which are already well-characterised) were eliminated from the study as not being novel, thus leaving a total of sixty one candidates (Supplementary Table S1). Based on the criteria of high level of gene expression, two of the sixty one tags were identified as potential candidates for promoter study. However, one of the two tags, with the sequence CATGTTGTTCCGTGTAGTACC and henceforth referred to as Tag-A, was not annotated and thus selected as a candidate for further study. The Tag-A was the second most highly represented tag in cv Banks at 30 days post anthesis (dpa) with high representation also at 20 dpa (Fig. 1a). A novel aspect of the Tag-A expressing gene is its strong differential expression between cvs Banks and Kite, with a single copy of this tag detected out of approximately 20,000 tags sampled in the cv Kite Long-SAGE 8 dpa library.

### Characterisation of the gene corresponding to the Tag-A

Tag-A was found to match with complete homology to a poorly characterised Unigene cluster Ta.2025 composed of four wheat EST sequences derived from mature/developing wheat seed (Supplementary Fig. S1). BlastN, MegaBlast and Discontiguous-MegaBlast analyses of Ta.2025 EST sequences yielded no homology to any known gene present within the NCBI non-redundant (nr) database. When one of the wheat EST sequence of the Ta.2025 Unigene cluster was compared to the NCBI EST sequence database (est_other) there was some homology, ranging from 34% to 61%, to twenty seven EST sequences of the Ta.40040 wheat Unigene cluster derived primarily from developing seed (Supplementary Fig. S2). Further attempts to detect homology with annotated sequences were based upon translated amino acid similarities, where the predicted protein sequences were based on the predicted open reading frame (ORF) from the four ESTs of the Ta.2025 cluster. However blastX and tblastx similarities with other protein sequences (~30%) were not high enough to assign a putative function with any degree of confidence. Tag-A also matched sixteen EST sequences from a cDNA library generated in our laboratory (Supplementary Fig. S3) using the same RNA isolated from 14 dpa developing seeds and used for LongSAGE. The four publicly available EST sequences, as shown in Supplementary Fig. S1, and the sixteen 14-dpa EST sequences, as shown in Supplementary Fig. 3, showed high homology to each other (Supplementary Fig. S4), and the resultant contig sequence had a putative open reading frame (ORF) and had a perfect match to the Tag-A sequence (Fig. 1b). The contig shown in Fig. 1b is hereafter referred to as ‘wheat bread making gene’ (*wbm* gene). The structure of the protein encoded by the *wbm* gene was determined using the “InterProScan” and the “Phobius” program from a publically available bioinformatics facility (EBI, http://www.ebi.ac.uk/Tools/pfa/phobius/). The *wbm* gene encodes a small protein where the open reading frame consists of a predicted signal peptide of 27 amino acid (aa) residues (Fig. 1b,c) and a non-cytoplasmic domain which spans from residue 28 to residue 75 (Fig. 1b). The mature wbm protein consists of 48 aa and included 5 glycine and 6 proline residues respectively. Also located on the mature peptide are four cysteine residues with a distribution pattern of CYS-(X = 7)-CYS-(X = 6)-CYS-(X = 1)-(CYS), where X represents any other aa residue. As outlined above, a putative function could not be assigned, the predicted ORF was found to contain an ML domain which is a MD-2-related lipid-recognition domain that is present in several proteins of unknown function in plants, animals and fungi. These proteins are predicted to mediate diverse biological functions through interaction with specific lipids. In addition, motif and structural prediction of the putative translated amino acid sequence via PredicNGS-TProtein indicates this gene probably encodes a small microbody-associated protein^16^.

### Expression profile of the *wbm* gene in developing seeds of wheat genotypes

**Figure 2:**
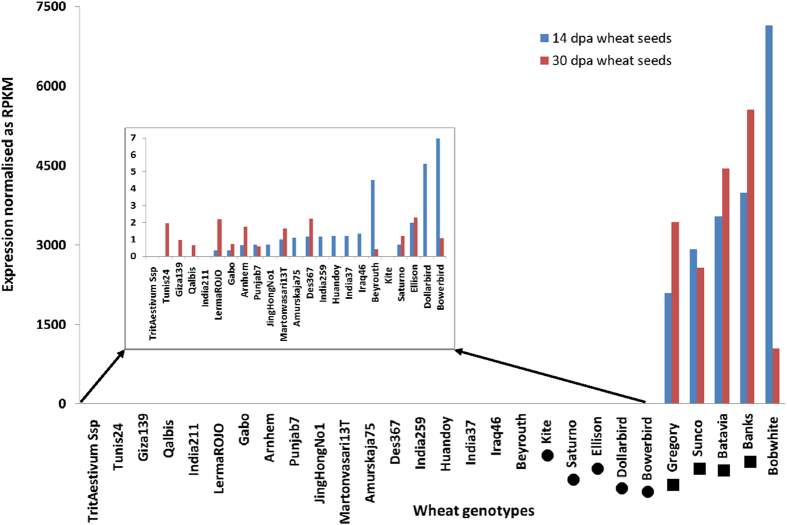
Transcript profile of the wheat bread making gene (*wbm)* gene in developing seeds of several wheat genotypes. Total RNA was extracted from whole developing seeds at 14 and 30 days post anthesis (dpa), cDNA prepared, and sequenced using an Illumina analyser. • and ■, genotypes known to be poor and good for breadmaking respectively. RNA-seq analysis was carried out using the TaGI sequences as the reference sequences. The *wbm* gene read counts for various genotypes were normalised as reads per kilobase per million reads mapped to TaGI. TaGI, *Triticum aestivum* gene indices is a list of tentative consensus cDNA sequences and can be accessed from DFCI http://compbio.dfci.harvard.edu/tgi/plant.html.

Data from RNA-seq analysis carried out at 14 dpa and at 30 dpa in developing wheat seeds was processed to obtain normalised *wbm* gene expression (Fig. 2). Data in Fig. 2 indicates that in most wheat genotypes, *wbm* gene expression was negligible at both the development stages except for that in cv. Gregory, Sunco, Batavia, Banks and Bobwhite. In cv. Sunco and Bobwhite, *wbm* gene expression was higher at 14 dpa than at 30 dpa, but was higher at 30 dpa than at 14 dpa for cv Gregory, Banks and Batavia. In addition, *wbm* gene expression, as a percentage of total gene expression, was high at both development stages (14 dpa and 30 dpa) in five genotypes; Gregory (0.18% and 0.28%), Sunco (0.24% and 0.21%), Batavia (0.29% and 0.35%), Banks (0.33% and 0.43%) and Bobwhite (0.54% and 0.08%). These genotypes with high expression all have good bread quality while the low expressing genotypes all have poor bread quality. The range in expression between these two groups was very large with many genotypes either showing very low expression (less than 10RPKM at 14 dpa) or very high (more than 1000RPKM at 14 dpa) (Fig. 2).

### Wheat genotype-specific predominant *wbm* sequence variants

RNA-seq analysis using the CLC-WB allowed the extraction of reads mapped to a target sequence. In this study, for each of the wheat genotypes and at each development stage (14 and 30 dpa), Illumina sequence reads that mapped to the *wbm* gene consensus sequence were extracted and used to obtain a genotype-specific Illumina-read-derived-consensus-*wbm*-cDNA sequence (Ilu-*wbm*-cDNA). The Ilu-*wbm*-cDNA sequence for genotypes with high *wbm* gene expression spanned the entire *wbm* gene contig region including the 5′ and 3′-UTR region. For those genotypes with low *wbm* gene expression, the corresponding Ilu-*wbm*-cDNA was not continuous due to low read coverage, and thus the sequence corresponded to the 5′-UTR, part of the coding region and the 3′UTR region.

**Figure 3:**
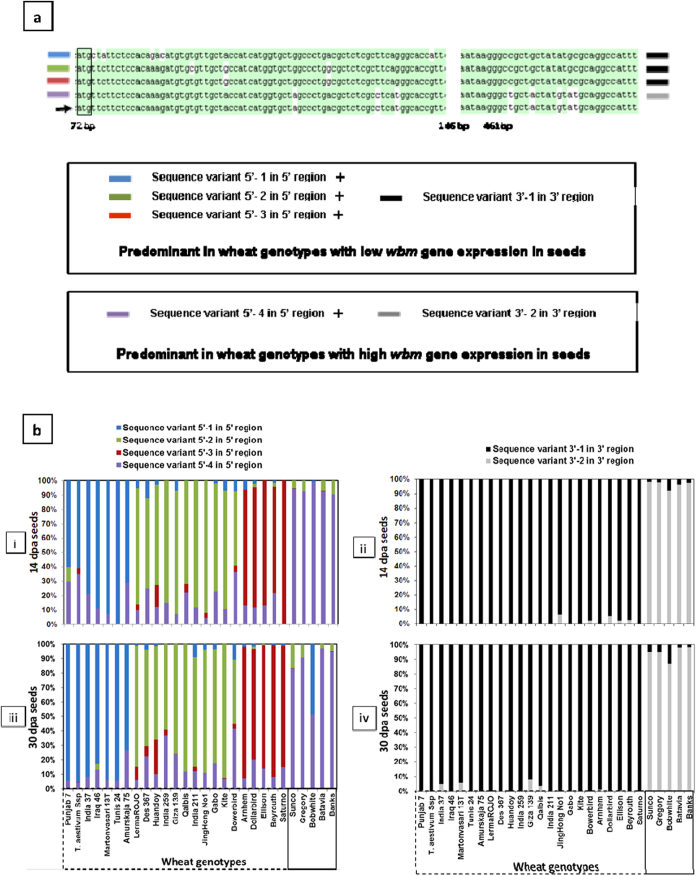
DNA sequence variation in the wheat bread making gene (*wbm*) and wheat genotype-specific expression of these variants. (**a**) cDNA sequence variants at the 5′ and 3′ ends of the CDS. Wheat genotypes showing low or high *wbm* gene expression had specific combinations of sequences in the 5′ and 3′ region of the consensus cDNA sequence. Base pair positions are in relation to the *wbm* contig sequence which is indicated by an arrow. The closed vertical box indicates the proposed ATG start site. Sequence variants at the 5′- and the 3′ regions and specific are shown in rectangular boxes. (**b**) Genotype-specific expression of the *wbm* sequence variants. Genotype-specific expression of each of the 5′ and 3′ sequence variants of the *wbm* gene for each genotype at 14 and 30 dpa is shown as a percentage of total normalised *wbm* gene expression within a genotype. Read counts were normalised with respect to total mapped reads. Days post anthesis (dpa).

The Ilu-*wbm*-cDNA sequence alignments for each of the genotypes at both 14 dpa and 30 dpa samples, around part of the 3′-UTR region and around the coding region at the 5′-end of the Ilu-*wbm*-cDNA led to the identification of genotype-specific and predominantly expressed specific sequence variants. The alignment of the 3′UTR regions of all the Ilu-*wbm*-cDNA indicates single nucleotide polymorphisms (SNPs), around positions 469-to-489 bp of the *wbm* gene contig region, leading to two haplotype sequences (Fig. 3). One of the haplotypes in the 3′-region is predominantly expressed in genotypes with low *wbm* gene expression and is hereby referred to as “Sequence variant 3′-1” while the other haplotype in the 3′-region is predominantly expressed in genotypes with high *wbm* gene expression and is hereby referred to as “Sequence variant 3′-2” (Figs. 2 and 3a).

The alignment of part of the coding regions of all the Ilu-*wbm*-cDNAs indicates SNPs, around positions 72-to-146 bp of the *wbm* gene contig, leading to four specific haplotypes (Fig. 3a) in the 5′-region. In genotypes with high *wbm* gene expression (Fig. 2), a single specific consensus haplotype was dominant and hereby referred to as “Sequence variant 5′-4” (Fig. 3a). In genotypes with low *wbm* gene expression (Fig. 2) three consensus haplotypes were dominant (Fig. 3a) and hereby referred to as “Sequence variant 5′-1”, “Sequence variant 5′-2” and “Sequence variant 5′-3”.

### Genotype-specific differential expression of the *wbm* gene variants

The contribution of the *wbm* sequence variants (Fig. 3a) to the total *wbm* gene expression in the wheat genotypes tested was plotted as an expression profile of the *wbm* sequence variants in the 5′-region and 3′-region (Fig. 3b). In most wheat genotypes tested and at both time points, the expression profile of the *wbm* sequence variants in the 5′ region indicates a general pattern with a predominant expression of one of the four sequence variants. However, in some of the wheat genotypes (14 and 30 dpa Bowerbird and 30 dpa Bobwhite) expression of one other *wbm* sequence variants in the 5′ region were not very much lower than the expression of the dominant sequence variant (Fig. 3b i and iii). In the genotype Bowerbird, the *wbm* sequence variants 5′-2 and 5′-4 are predominantly expressed at both time points. In the genotype Bobwhite, the sequence variant 5′-4 is predominantly expressed at 14 dpa with negligible expression of sequence variant 5′-1, and this expression pattern changes at 30 dpa with a reduction in expression of sequence variant 5′-4 and a huge increase in expression of sequence variant 5′-1. The expression of the *wbm* sequence variants in the 3′-region indicates the dominant expression of *wbm* sequence variant 3′-1 and of variant 3′-2 in wheat genotypes with low *wbm* expression and high *wbm* expression respectively (Fig. 3b ii and iv). Comparing the transcript profile based on the *wbm* sequence variants at the 5′ and the 3′-region, it can be inferred that the *wbm* sequence variant 3′-1 in the 3′-region is predominantly associated with sequence variants 5′-1, 5′-2 and 5′-3 in the 5′-region, while the *wbm* sequence variant 3′-2 in the 3′-region is predominantly associated with sequence variant 5′-4 in the 5′-region.

### Isolation and sequence-structure of the 5′-upstream region of the *wbm* gene

Inverse PCR by “Genome Walking” was used to isolate the 5′-upstream regions of the *wbm* gene. Four Genome Walker (GW) libraries generated using DNA from wheat genotypess (cvs) Banks and Kite, were used as template in a PCR reaction and this generated several fragments of varying lengths (Supplementary Fig. S5i). Alignments of Banks and Kite GW fragments indicate several sequence variants (*GWseqVar*) with regions of high homology immediately at the 3′-end but reduced homology progressively towards the 5′-end (Supplementary Fig. S5ii). The alignment data also indicates the presence of genotype-specific unique and common sequences variants; where *GWseqVar-3* and *GWseqVar-6* are present only in Banks, *GWseqVar-5* is present only in Kite, while *GWseqVar-*1, *GWseqVar-*2 and *GWseqVar-*4 are present in both genotypes. Data in (Supplementary Fig. S5iii) also indicates the presence of at least three GWseqVar groups identified as *wbm*-A, *wbm*B and *wbm*-C. Group *wbm*-A is made up of four subgroups consisting of *GWseqVar-*1, *GWseqVar-*3, *GWseqVar-*4 and *GWseqVar-*5, *wbm*-B consists of *GWseqVar-6*, and *wbm*-C consists of *GWseqVar-2*.

### PCR to determine presence of GWseqVar of the *wbm* gene in Banks, Kite and Bobwhite

PCR Amplified fragments using primers annealing to specific regions of the various *GWseqVariants-*1 to *GWseqVariants-*6 but not *GWseqVariants-*5 are shown in Supplementary Fig. S6. A 918 bp fragment corresponding to *GWseqVar-1* was amplified in all three genotypes. A 662 bp fragment corresponding to *GWseqVar-2* was amplified in Banks and Kite but not Bobwhite. A 961 bp fragment corresponding to *GWseqVar-3* was amplified in Banks and Bobwhite but not Kite. A 509 bp fragment corresponding to *GWseqVar-4* was amplified in Banks and Kite but not Bobwhite. A 135 bp fragment corresponding to *GWseqVar-6* was amplified in all three genotypes. Attempts to amplify regions of the *GWseqVar-5* using three different primer combinations failed in all three genotypes.

### Relationship between various wheat genotypes for the expression of the wheat breadmaking gene (*wbm)* gene in developing seeds, bread making quality and the presence of the 5′-upstream GWseqVar-3 of the *wbm* gene

**Figure 4:**
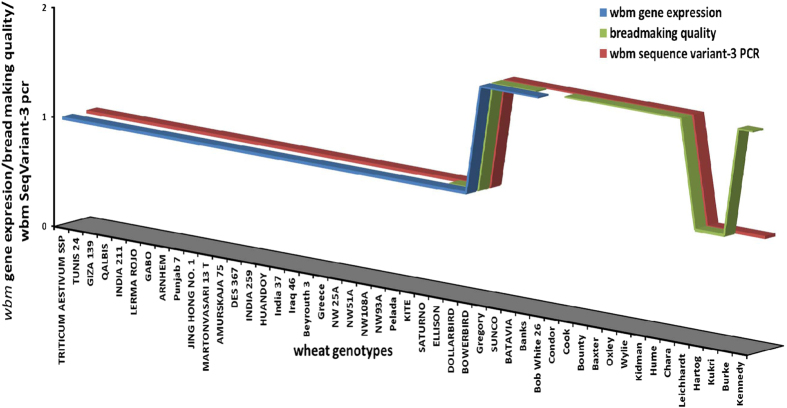
Relationship of various wheat genotypes for expression of the wheat breadmaking gene (*wbm)* gene in developing seeds, bread making quality and the presence of the 5′-upstream GWseqVar-3 of the *wbm* gene . X axis scale of 1 or 2 correspond to low or high *wbm* gene expression respectively, good or poor bread making quality respectively, absence or presence of the *GWseqVar-3* 5′-upstream region of the *wbm* gene respectively. Total w*bm* gene expression was determined by next generation sequencing. Good or poor breadmaking quality data for wheat genotypes was obtained from reports published by the Australian wheat Industry organisations. The presence of the *GWseqVar-3* of the 5′-upsteam region of the *wbm* gene was determined by allele-specific PCR. It can be seen that for the first 34 genotypes, there is an association between *wbm* gene expression and the 5′-upstream *GWseqVar-3* variant of the *wbm* gene. (For the first 27 genotypes the GWseqVar-3 variant is absent and there is low *wbm* gene expression, whilst for the next seven genotypes the GWseqVar-3 variant is present and there is high *wbm* gene expression). Low expression of the *wbm* gene is associated with poor breadmaking quality and the absence of the *GWseqVar-3* variant of the *wbm* gene. The same association between good breadmaking quality and the presence of the *GWseqVar-3* variant of the *wbm* gene was observed for an additional set of wheat genotypes Condor to Kukri, except for the genotypes Burke and Kennedy. Gene expression data for the additional set of wheat genotypes (Condor to Kennedy) has not been obtained.

The wheat genotypes taken for RNA-seq analysis (Fig. 2) were subjected to PCR analysis to determine the presence of the *GWseqVar-3* promoter sequence. The PCR band corresponding to *GWseqVar-3* specific was present in all genotypes with high *wbm* gene expression but was absent in all those genotypes with low *wbm* gene expression (Fig. 4). When the PCR analysis was applied to an expanded set of genotypes known for their breadmaking quality the same association between a positive PCR for the*GWseqVar-3* allele and good breadmaking quality was observed except for the genotypes Burke and Kennedy (Fig. 4). The level of expression of the *wbm* gene and the sequence of the promoter region is not know for these genotypes.

### Expression profile of the *wbm gene* in aleurone and starchy endosperm of Banks

**Figure 5:**
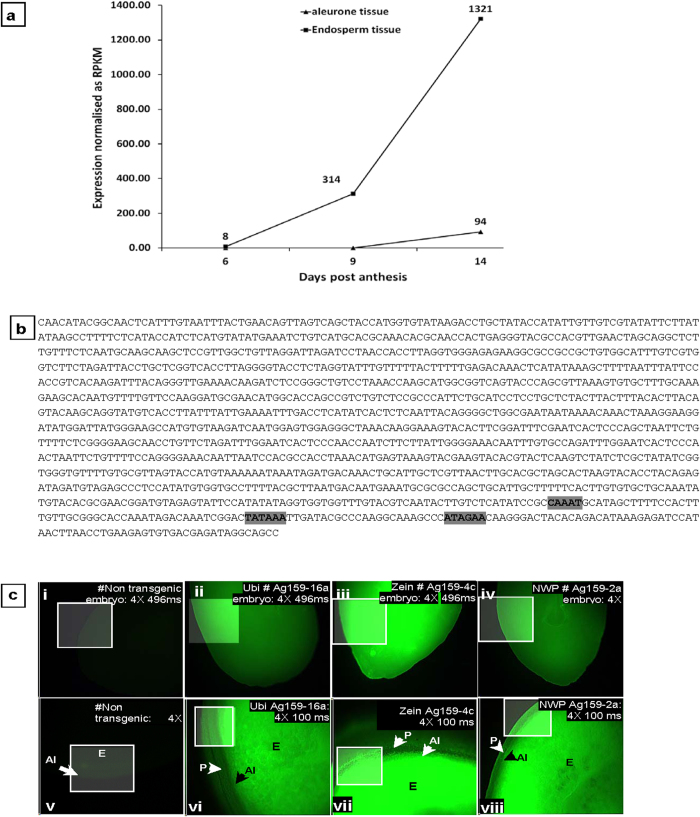
Transcript profile and characterisation of the 5′-upstream promoter variant-3 of the *wheat bread making (wbm)* gene in wheat cv Banks and its transgenic expression in maize seed. **a,** A comparison of the expression of the *wbm* gene in aleurone versus endosperm of the seed of wheat genotype cv. Banks. **b,** Nucleotide sequence of the 1379 bp 5′-upstream region of the wheat bread making promoter sequence variant-3 (*wbmp*-BA3) from wheat cv Banks. Putative TATA and CAAT boxes are shown as highlighted text. ATAGAA, a putative region for transcription initiation; **c,** Green fluorescent protein gene expression by the ubiquitin, zein and *wbmp*-BA3 promoters in developing seed tissues of transgenic maize plants. (i,ii,iii,iv), embryo; (v,vi,vii,viii), transverse section of developing seed at 20-25 days after flowering; (i,v), no transformed tissue, (ii,vi), ubiquitin promoter-transformed tissue; (iii,vii), zein promoter-transformed tissue; (iv,viii) *wbmp*-BA3 promoter-transformed tissue. The *ubi*, *zein* and the *wbmp*-BA3 promoters direct the expression of GFP in the embryo, aleurone and the endosperm tissue. Detecting the expression of GFP in transgenic tissue was carried out by comparison with corresponding tissues of non-transformed plants (i,v). Observations were carried out under blue light (excitation, 489 nm; emission, 510 nm) using a compound fluorescence microscope. Representative images for promoter lines are shown and images were taken at different exposure times for clarity of images. #, line numbers of independent transgenic events; s, seconds; ms, milliseconds. Enhanced areas of images are represented as a box with a white border. Some of the composite figures may not be labelled to avoid repetition.

Using next generation sequencing, profiling for the *wbm* transcript in developing aleurone and starchy endosperm tissues of the wheat genotype Banks indicates the *wbm* gene is predominantly expressed in the starchy endosperm tissue (Fig. 5a). In the aleurone tissue, transcript reads corresponding to the *wbm* gene were close to zero at 6 dpa and 9 dpa with a slight increase at 14 dpa. In the starchy endosperm tissue, transcripts corresponding to the *wbm* gene were detected at 6 dpa which increased slightly at 9 dpa and further at 14 dpa. This study was not carried out using 30 dpa samples due to the difficulty in complete separation of aleurone from starchy endosperm tissue.

### Sequence and structure of the *GWseqVar-3* 5′-upstream of the *wbm* gene from Banks

As outlined above we identified two unique sequences of the 5-upstream region (promoter regions) of the *wbm* gene in the wheat genotype Banks. These 5′-upstream region of two *wbm* genes were 1375 bp and 473 bp fragments and corresponded to *GWseqVar-3* and *GWseqVar-6* respectively. The 1375 bp 5′-upstream promoter fragment corresponding to *GWseqVar-3* was chosen for further study as it was the longer of the two fragments. The sequence of *GWseqVar-3* with putative “TATA-box” and “CAAT-box” sites are shown in Fig 5b. The sequence of the *wbm* promoter corresponding to the *GWseqVar-3* is henceforth referred to as *wbmp*-BA3.

### Activity of maize Ubiquitin, Zein and wbmp-BA3 promoters in transgenic maize

A number of transgenic maize plants were derived from independent *Agrobacterium*-inoculation experiments with binary constructs containing the maize ubiquitin promoter or the maize zein promoter or the *wbmp*-BA3 promoter linked to the green fluorescent protein gene (*gfp*). The ubiquitin (*ubi*) promoter drives constitutive expression of GFP in both seed and non-seed tissues (Fig. 5c ii,iv and Supplementary Fig. S7 b,f), and images from these plants were used as positive controls to compare expression of GFP in corresponding tissues of *wbm*-promoter transformed and non-transformed tissues.

Both the zein-promoter and the *wbmp*-BA3 promoters direct seed-specific expression of GFP with no expression of GFP detected in leaf or the root tissue of maize (Supplementary Fig. S7 c,g and d,h). In developing seed tissue at 20 DAF, both the zein and the *wbmp*-BA3 promoters, direct expression of GFP only in the embryo, the aleurone and the endosperm tissues as shown in Fig. 5c iii,vii and iv and vii respectively, with similar results obtained for seeds at 30 dpa.

### Quantative analysis of promoter activity in T_1_-generation maize seed tissue

The wheat *wbmp*-BA3, maize *zein* and maize *ubiquitin* promoter-directed GFP expression in eight mature dry T_1_-generation seeds (T_1_-seeds) was quantified by measurement of GFP protein by enzyme-linked immunosorbent assay (ELISA) (GFP-ELISA) and is shown in (Supplementary Fig. S8). Those seeds showing no GFP concentration are not plotted in Supplementary Fig. S8 as these represent null seeds (segregating without transgene loci) or transgenic seeds with GFP concentration below the sensitivity range of the ELISA. The results of the GFP-ELISA for the three promoters indicate a variation in the expression of GFP in the T_1_-seeds. Of all the promoter lines tested, the amount of GFP protein was higher in lines corresponding to the maize *ubiquitin* promoter. The range of GFP protein expressed under the wheat *wbmp*-BA3 promoter and the maize *zein* promoter was under 1 ug/mg of total extracted seed protein. Within a promoter group the highest GFP accumulation per seed corresponded to 0.05% of soluble seed protein for the wheat *wbmp*-BA3 promoter, 0.09% for the maize *zein* promoter and 0.49% for the maize *ubiquitin* promoter (Supplementary Fig. S8).

### Location of the *wbm* gene in the wheat genome

We used BLAST to seach for the *wbm* gene in the Ensemble (www.plants.ensembl.org, searched 24 February, 2015) wheat genome assembly. The gene was located on chromosome 7 L but had not been annotated. No other wheat quality genes are located in this part of the genome^17^. The apparent rarety of the high expressing allele in the wheat gene pool means that it is unlikey to have been in wheat populations included in earlier studies.

## Discussion

Wheat is one of the most important cereal crops in the world with world production around 650 million tonnes with much consumption in the form of a variety of breads across different countries and cultures. Growing global demand for wheat requires ongoing genetic improvement. However new wheat varieties must retain the essential quality characteristics of wheat. Assessing a wheat genotype for bread making quality in a baking test requires a large sample of wheat, a requirement that is prohibitive in applying this test in the early stages of selection of wheat lines in wheat breeding^18^. Bread quality is not well correlated with other measures of wheat quality such as flour milling yield and water absorption or dough qualities such as dough strength, extensibility and mixing characteristics^19^,^20^,^21^,^22^. A lack of understanding of the biochemical and molecular genetic control of bread making quality has prevented the application of molecular tools to supporting accelerated wheat breeding. Selection for the alleles resulting in high expression of the *wbm* gene may prove an important option that can be used in addressing food security by reducing the constraint of bread quality in increasing wheat yields globally.

The *wbm* gene expression is tissue-specific and highly differential in wheat genotypes. In developing wheat seeds of cv Banks, SAGE analysis indicated that the *wbm* gene is expressed as early as 8 dpa and increased steadily till 30 dpa and this pattern of gene expression was confirmed by RNA-Seq analysis (Figs. 1a,2). In addition, the RNA-Seq analysis indicates the *wbm* gene expression to be starchy-endosperm-specific (Fig. 5a). Although, *wbm* gene expression in the aleurone was observed, it was less than one-tenth of the endosperm *wbm* gene expression and this observation could be an artefact resulting from the difficulty in the complete separation of the starch endosperm from the aleurone tissue of 14 dpa developing seeds (Fig. 5a). The *wbm* gene is differentially expressed in cvs Banks and Kite where high expression was detected in cv Banks but not in cv Kite by SAGE analysis, but low expression was detected in Kite by RNA-Seq analysis indicating the high sensitivity of the NGS and RNA-Seq analysis as compared to traditional LongSAGE analysis. We conducted RNA-Seq analysis on an expanded set of 28 wheat genotypes (and also in Kite and Banks) and determined that the *wbm* gene was differentially expressed in developing seeds, with high *wbm* gene expression observed in cvs Gregory, Sunco, Batavia, Banks and Bobwhite in both 14 dpa and in 30 dpa developing seeds (Fig. 2). The RNA-Seq analysis also revealed the presence of several sequence variants of the *wbm* gene which is in agreement with a number of cDNA sequence variants isolated in our laboratory from the cv Banks. In addition, the RNA-Seq analysis revealed a predominant *wbm* gene sequence variant expressed in each of the wheat genotype, which when aligned for all the wheat genotypes indicated genotype-specific selective predominant expression of a *wbm* gene sequence variant. Wheat genotypes showing low or high *wbm* gene expression had specific combinations of sequence variants in the 5′ and 3′ region of the predominantly expressed *wbm* cDNA sequence (Fig. 3a). Three sequence variants of the *wbm* gene, each of which was found to be predominantly expressed in wheat genotypes with low *wbm* gene expression. However, one sequence variant combination of the *wbm* gene, sequence variant 5′-4 in 5′ region and sequence variant 3′-2 in 3′ region, was found to be predominantly expressed in all wheat genotypes with high *wbm* gene expression including in the cv Banks (Fig. 3b).

Expression of the specific sequence variant of the *wbm* gene is probably controlled at the transcriptional level and is of a rare lineage. Isolation and alignment of the 5′-upstream sequences of the *wbm* gene in cvs Kite and Banks indicated the presence of six sequences variants which are common and unique to both these genotypes (Supplementary Fig. S5). PCR with primers designed to specifically amplify and discriminate products based on their size, confirmed the Genome Walker results (Supplementary Fig. S6), where GWseqVar3 is unique to Banks and GWseqVar1, GWseqVar2 and GWseqVar4 are present in both Banks and Kite. We were unable to amplify GWseqVar5 sequence from Kite and in addition, although the GWseqVar6 sequence was isolated from Kite and not fromBanks, the PCR results indicated that this variant is not unique to Banks as it is amplified in both Kite and Banks (Supplementary Fig. S6). The GWseqVar3 sequence variant, which is unique to Banks but not to Kite, was also amplified in all wheat genotypes with high *wbm* gene expression (Fig. 4 and Supplementary Fig. S6), suggesting that this sequence variant of the 5′-region of the *wbm* gene contributes in the transcriptional regulation of the high *wbm* gene expression in selected wheat genotypes. As wheat is a ployploid species, we investigated to determine the wheat progenitor lineage of the variants of the *wbm* gene and possibly assign them as paralogues or orthologues. PCR for the six variants when carried out on a number of genotypes corresponding to wheat progenitor species indicated the amplification of all the GWseqVar-specific bands in the wheat progenitor genotypes , but we could not assign each of the sequence variants to be orthologues or paralogues. However, the amplification of the GWseqVar-3 sequence in a small number (5%) of *T. monococcum* (11 lines tested) and *T.urartu* samples (22 lines tested) but not in *T. turgidum* (AABB) (62 lines tested) genotypes. As *T.tauchii* genotypes were not tested it is possible that the GWseqVar-3 is of a rare linage which has introgressed in hexaploid wheat from the wheat progenitor contributing the AA- or the DD-genome type.

The close association between high *wbm* gene expression and the presence of the GWseqVar3 sequence variant in the wheat genotypes tested (Figs. 2,4) is interesting. Data for a number of grain quality traits, for the wheat genotypes tested, such as milling quality, grains size, grain hardness using Single Kernel Characterisation System (SKCS) analysis and protein content, failed to correlate with wheat genotypes having high or low wbm gene expression. However, the wheat genotypes with high *wbm* gene expression (high-*wbm*-expressers), Sunco, Gregory, Batavia and Banks, are all known for their superior breadmaking quality. In addition, we also determined that all of the high-*wbm*-expressers had the wheat genotype WW-15 or genotypes derived thereof in their pedigree. The wheat genotype WW-15, a released genotype in Australia, was used to breed cv Condor known for good bread making. The wheat cv Cook and Banks are also known for their good bread making quality and were both derived from Condor. Another genotype Janz which was bred from Cook and Condor, is also known for its good bread making quality. The cv Condor is known to have been used as a common parent in CIMMYT wheat programs thus leading to the possibility that several modern CIMMYT wheats may be high-*wbm*-expressers and good for bread making. To test the hypothesis that good breadmaking is associated with high *wbm* gene expression, we applied the PCR test to an expanded set of wheat genotypes. The strong association between high *wbm* gene expression and the presence of the GWseqVar3 sequence variant, which could be checked by a simple PCR, allowed us to use this PCR test instead of expression profiling on the second expanded set of wheat genotypes. We selected fourteen wheat genotypes with good or poor breadmaking quality and with or without WW-15/Condor/Cook as parents in their pedigree. The GWseqVar3 sequence variant when present was always associated with good bread making genotypes and these had WW-15 in their pedigree, such as Condor (WW 80/2*WW-15), Cook (Timgalen/QS 7165//Condor), Janz (3-AG-3/Condor//Cook), Baxter (QT2327/Cook//QT2804), Oxyley (derived from WW-15), Wylie (QT2327/Cook//QT2804), Chara (derived from Cook). The GWseqVar3 sequence variant when also present in cvs Bounty (Leichhardt//Batavia), Kidman and Hume (Pelsart//Batavia), all good for bread making, and were derived from Batavia (which was derived from Banks) also a high *wbm* gene expresser and good for bread making. It is interesting to note that the cross between Pelsart//Batavia, both good for bread making and having the WW15 background, led to the generation and release of an unusually high number of wheats as genotypes including cvs Gregory, Kidman and Hume with good bread making properties. Although breeders were perplexed why the Pelsart//Batavia cross lead to so many wheat lines good for bread making, it is now clear that presence of the GWseqVar3 sequence variant in both parents meant that all progenies would have been good for breadmaking due to the presence of the GWseqVar3. This work on the *wbm* gene reveals that both Pelsart and Batavia are the key as Pelsart is derived from Condor, Batavia is derived from Banks which is derived from Condor, and Condor is derived from WW-15. The analysis on the second-expanded set of wheat genotypes also included genotypes with no WW-15 background, but belonging to the Hartog or Pavon family. Hartog is known to be good for yellow alkaline noodle (YAN) but marginal for breadmaking. The genotypes Diamondbird, Arnhem and Leichhardt (Fig. 2), all from the Pavon/Hartog derived families are negative for the GWseqVar3 sequence variant and are also poor or marginal for breadmaking. All genotypes with a high level of expression had good quality and all with a low level of expression had poor quality. However, cvs Burke and Kennedy found to be negative for the PCR for the GWseqVar3 sequence variant are good for breadmaking but the level of expression of the gene in these genotypes is not known. They may contain a different promoter mutation that results in high expression or may have other mechanisms of achieving good bread quality. Thus, this PCR analysis for the GWseqVar3 sequence variant demonstrates that when genotypes are positive for the GWseqVar3 sequence variant they are also good for breadmaking. The close association between GWseqVar3 sequence variant and good breadmaking can be used as a marker for the early selection of wheat lines in a wheat breeding program. The *wbm* gene expression is high in cv Bobwhite-26 (Fig. 2), and it would be interesting to identify the lineage contributing to this high expression as WW-15 is not present in its pedigree. The increased *wbm* gene expression in Bobwhite-26 may not lead to good breadmaking as this genotype has the T1BL.1RS translocation from rye derived from Averora which is one of its parents in its pedigree (Averora//Kalyansona/Bluebird//Woodpecker) and this translocation is associated with the sticky dough character^23^.

The *wbm* gene encodes a small mature peptide with molecular weight ca 5000, is comprised of 48 aa residues, but is unlike the purothionins or the prolamins group of proteins in both amino acid composition and structure. Composition of the wbm protein indicates the absence of glutamic acid, the presence of glycine at 10.4% and proline and glutamine each at 9.3%. The wheat purothionins are small single chains proteins with molecular weight of around 6000 (45 amino acids in length) and present as lipid complexes in the endosperm^24^. Purothionins are rich in cysteine and lysine, and also unlike the wbm and other wheat proteins, are low in glycine and proline. The purothionins in wheat are known to be in the top 30 highly expressed in the wheat endosperm, but their biological function of the purothionins in wheat is not known^25^. Wheat purothionins are known to be toxic to some strains of brewer’s yeast and animal cells when tested *in vitro*^26^,^27^. Although the purothionins are rich in cysteine and result in intermolecular disulphide bonds^5^, this interaction was thought to play a role in breadmaking. However, de-fatted and reconstituted flour or fortifying aged flour known to have reduced levels of purothionins and baking quality, failed to have any impact on loaf volumes^28^,^29^. The wbm gene has poor homology to all of the known purothionins genes from wheat (data not shown).

The amino acid composition of the wbm protein is also unlike the prolamins group of storage proteins in cereals which are rich in glutamic acid, glutamine and proline^32^,^33^, The *wbm* gene encodes a protein that does contain four cysteine residues but its distribution pattern of -Cys-(X = 7)-Cys-(X = 6)-Cys-X-Cys-, differs to the characteristic pattern, Cys-(X = 7-13)-Cys-(X = 8-26)-Cys-Cys-(X = 8-30)-Cys-X-Cys-(X = 20-48)-Cys, of cysteine residues present in all prolamins group of proteins^32^. The prolamins in wheat which represent the storage proteins and account for 35% of the total grain protein and are comprised of the monomeric gliadens and high molecular weight (HMW) and low molecular weight (LMW) polymeric glutenins^33^. The cysteine residues in both the gliadens and glutenins play a role in inter and intra molecular disulphide bonds. The role cysteine residues play in dough properties is extensively studies for the HMW glutenins than the gliadens, resulting in the identification of a number of HMW alleles that contribute to, in part at least, to bread making 32,33,34,35,36. Although the cysteine residues, in the HMW and LMW subunits, may interact to form di-sulphide bonds, it is the interplay of cysteine bonds in the dough which ultimately leads to the end product with the desired property^31^. The presence of 4 cysteine residues in the predicted wbm protein could lead to intra-molecular disulphide bonds or intermolecular disulphide bonds with other wbm units or other prolamins proteins. The number of cysteine residues and their specific location on different alleles of HMW units are thought to play a role in the dough properties and breadmaking^32^,^37^,^38^,^39^. The extra cysteine residue in the 1DX5 allele of the LMW subunit was suggested to play a role in dough property leading to weakening of the dough^38^,^40^. However, overexpression of the DX5 allele in transgenic wheat led to incomplete mixing of the flour due to strong dough formation and leading to reduced loaf volume, thus giving the opposite result^5^,^41^,^42^. In addition, overexpression of a number of other HMW alleles in transgenic wheat has led to opposite effects on dough properties^43^,^44^,^45^,^46^. These transgenic experiment results thus demonstrate the complex interplay of a number of other determinants, protein and non-protein, which collectively contribute to favourable dough characteristics and bread properties. It is important to note that in some wheat genotypes, the high expression of some HMW allelic forms than other subunits is thought to contribute to high dough strength^47^,^48^,^49^.

The association of good breadmaking wheat genotypes to high *wbm* gene expression and possibly increased wbm protein expression, opens the possibility of utilising classical breeding or transgenic approaches in converting poor breadmaking wheat into good breadmaking wheats. Wheat lines yielding as much as 30% more than any of the commercial genotypes are currently not used commercially for human consumption due to their poor breadmaking quality (Banks, P. unpublished). Using a classical breeding approach, a simple and reliable DNA-based PCR test, using DNA isolated from leaf tissue, can be used to select wheat lines with the desired GWseqVar3 sequence to ensure high expression of the *wbm* gene and good breadmaking quality. All genotypes displaying a high level of expression had good bread quality and diversity in the promoter sequences may explain the differnces in expression levels observed.

## Experimental

### Plant Material

Seeds of wheat genotypes were sourced from the Australian Winter Cereal Collection, Tamworth, Australia. Seeds were germinated either in a glasshouse or in a growth cabinet with 12 hrs of light and at day and night temperatures of 20 °C and 18 °C respectively. Seeds corresponding to 14 and 30 days post anthesis (dpa) seeds were collected as follows. Plants were tagged when awns were first visible at the flag leaf sheath as follows; date of awn observance, date of anthesis as +4days from date of awn observance, dates for 14 and 30 days post anthesis as +14 and +30 days from date of anthesis respectively. Furthermore, spikes ready for harvest (based on 14 or 30 dpa tag dates) were checked if the immature embryo corresponded to a 14 dpa or 30 dpa immature embryos as follows. A developing seeds was harvested and the embryo was gently excised and visually observed to match a 14 or 30 dpa immature embryos. Spikes were harvested and the top and bottom half from the centre of the spike was cut off and discarded while the rest of the spike was then snap frozen in liquid nitrogen. While under liquid nitrogen, the developing seeds from four or five spikes were then separated and stored at -70 C until pulverised using a tissue lyser (Qiagen, USA)^50^ and processed for RNA isolation.

### Wheat genotypes good and poor for breadmaking

Australian wheats are graded and classified for end use by Wheat Classification Council, which is comprised of a committee to the Board of Wheat Quality Australia. Wheat Quality Australia relies on data generated from the National Variety Trials (NVT) to classify new varieties. We obtained data pertaining to wheat genotypes classified as good or poor for breadmaking based on annual NVT data released by the Grains Research and Development Corporation (GRDC)^51^, and can be accessed from the GRDC and DEEDI website^53^,^52^. Industry ratings of wheat genotypes as good, acceptable or excellent for bread making was used to identify and group genotypes in this study as good for bread making. Similarly, wheat genotypes with industry rating of poor or marginal for bread making baking was used to identify and group genotypes in this study as poor for bread making.

### LongSAGE to identify genes with high gene expression

Long-SAGE libraries of wheat seed (Triticum aestivum cv. Banks and Kite) were constructed and published elsewhere^54^. Genotypess Banks and Kite were selected as these are known examples of good and poor quality bread wheats respectively. Essentially, pooled seed consisted of samples at the 8, 14, 20, 30 day post anthesis stages and also at 40 dpa (considered as mature seed). The present study sought to define the most abundant transcripts for the 20 and 30 dpa stages (combined) and then determine the most suitable candidate(s) for further gene promoter studies, with the intention of developing the candidate as a tool for directing desired expression of transgene/s in transgenic plants. Tags were sorted based on increasing order of abundance, and the first one hundred abundant SAGE tags were subsequently annotated via BlastN comparisons with Genbank EST sequences and verified against results generated using the HarvEST sequence clusters.

### Isolation of aleurone and endosperm tissue, RNA isolation, cDNA construction and Illumina sequencing

Aleurone and endosperm tissue were isolated from wheat from cvs Banks and Kite and all steps from isolation of samples, cDNA preparation, Illumina library preparation and NGS sequencing and the raw data are as described previously^15^.

### RNA isolation

Total RNA was isolated using the Trizol Reagent (Invitrogen, Carlsbad, USA) as published elsewhere^52^. Total RNA quality and concentration were determined using the RNA 6000 Pico kit (Agilent, Santa Clara, USA) on a 2100 Bioanalyzer (Agilent Technologies, Inc, Santa Clara, CA, USA).

### NGS Sequencing

Sequencing was conducted by Southern Cross Plant Genomics, Lismore, Australia, on the Illumina GAIIx system and all steps were followed as per the manufacturer’s recommendation. NGS sequencing of cDNA from aleurone and endosperm tissue was carried out to obtain 75 bp paired reads, while all other NGS sequencing was carried out to obtain 100 bp paired end reads.

### Identification of gene corresponding to Tag-A

The sequence corresponding to Tag-A (CATGTTGTTCCGTGTAGTACC) was subjected to BLAST analysis to Unigenes in NCBI. Identified Unigene cluster sequences were then subjected to BlastN, MegaBlast and Discontiguous-MegaBlast analysis to the non-redundant nucleotide database in NCBI, and to NCBI ET sequences. Tag-A was also subjected for homology analysis to an a cDNA library prepared in our laboratory using the same RNA isolated from 14 dpa developing seeds of cv banks and used for the LongSAGE experiment.

### RNA-Seq analysis for expression of the gene corresponding to Tag-A

All NGS data (as 75 bp and as 100 bp paired end reads) was imported into CLC Genomics Workbench (CLC-GW) ver 7.0.4 (CLC Bio, Aarhus, Denmark) and trimmed using default parameter. Trimmed sequences were processed for RNA-Seq analysis using the RNA-Seq tool within the CLC-GW platform with the unannotated Triticum aestivum Gene Index (TaGI) used as reference sequences/database. TaGI consists of 221,925 tentative consensus sequences (TC) and derived from the DFCI Release 12.0 (The Computational Biology and Functional Genomics Laboratory, Dana Farber Cancer Institute and Harvard School of Public Health). Mapping parameters used were 0.9 for minimum length fraction, 0.8 for minimum similarity fraction and selecting the “include broken pairs” counting scheme. Expression values for each gene was normalised as Reads per Kilobase per million reads mapped to DFCI (RPKM). The EST contig corresponding to Tag-A, as identified by NCBI searches, when subjected to BLAST on DFCI showed homology to one TC in TaGI and corresponded to TC420043. Expression of the gene corresponding to Tag-A (TC420043) using RNA-Seq analysis was thus determined by extracting and counting reads which mapped to the tentative consensus TC420043 in TaGI. Statistical analysis of the RNA seq data to identify differentially expressed genes was carried out using the “Emphirical analysis of Differential Gene Expression” 55,56 a tool within the CLC-WB, with tag-wise dispersions estimated selected and p-values corrected for false discovery rate.

### General sequence analysis

All basic sequence analysis was carried out using Clone Manager 9 (Sci-Ed, Cary, NC, USA) and Chromas Pro (Technelysium, Qld, Australia).

### DNA isolation

DNA was isolated from 10 to 15 day old seedlings of wheat according to a published method^57^.

### Isolation of the 5′-upstream region of the wbm genes

Inverse PCR by “Genome Walking” was used to isolate the 5′-upstream regions of the NW gene using the Universal Genome Walker kit (Clontech, USA). Four Genome Walker (GW) libraries generated using DNA from wheat cvs Banks and Kite, were used as template in a PCR reaction. Primers combinations of AP1 (supplied with kit) and NW1 (5′ AGGTGGCCGGCGTCAACGGTGCCATGA3′) and AP2 (supplied with kit) and NW2 (5′ GGCTAGCACCATGATGGTAGCAACAC3′) were used in the primary and nested PCR respectively. Selected amplified fragments were cloned into pGEMT-easy vector (Promega, USA) and plated on ampicillin resistant plates according to the manufacturer’s recommendation. Using blue/white screening, five colonies derived from cloning each PCR fragment were selected for plasmid preparation followed by sequencing using M13F/M13R primers as recommended by the manufacturer.

### PCR to detect presence of the *wbm* allele

PCR screening of plants was carried out using purified genomic DNA from seedling/leaf tissue using NWPFor (CCGTCACAAGATTTACAGGGTTG) and NWPRev (TTATGGATCTCTTTATGTCTGTGT) primer pairs to generate a 961 bp fragment. PCR reaction for 10 cycles commenced with denaturing at 94 °C for 30 s, followed by annealing at 45 °C for 30 s and extension at 72 °C for 2 min and then for 25 cycles with denaturing at 94 °C for 30 s, followed by annealing at 50 °C for 30 s and extension at 72 °C for 2 min.

### Transformation of maize

Maize transformation was carried out by Agrobacterium-mediated transformation^58^.

## Additional Information

**How to cite this article**: Furtado, A. *et al.* A novel highly differentially expressed gene in wheat endosperm associated with bread quality. *Sci. Rep.* **5**, 10446; doi: 10.1038/srep10446 (2015).

## Supplementary Material

Supporting Information
