# Supplementary material for: A novel highly differentially expressed gene in wheat endosperm associated with bread quality
Source: Sci Rep. 2015 May 26;5:10446. doi: 10.1038/srep10446 (PMC4650634; doi:10.1038/srep10446)
Supplement: Supporting Information [file srep10446-s1.doc]

**Supplementary Figures and Tables**

A novel highly differentially expressed gene in wheat endosperm associated with bread quality

Furtado, A., Bundock, P.C., Banks, P.M., Fox, G., Yin, X. and Henry, R.J.

**a**

Ta#S12922787 1 ataaagagatccataacttaacctgaagagtgtgacgagataggcagcc**ATG**ttcttctccacaaagatgtgtgttgctaccatcatggtgctagccctgacgctctcgc

Ta#S13023380 1 ataaagagatccataacttaacctgaagagtgtgacgagataggcagccatgttcttctccacaaagatgtgtgttgctaccatcatggtgctagccctgacgctctcgc

Ta#S12922811 1 ---aagagatccataacttaacctgaagagtgtgacgagataggcagccatgttcttctccacaaagatgtgtgttgctaccatcatggtgctagccctgacgctctcgc

Ta#S13023458 1 ---aagagatccataacttaacctgaagagtgtgacgagataggcagccatgttcttctccacaaagatgtgtgttgctaccatcatggtgctagccctgacgctctcgc

TAG-A --------------------------------------------------------------------------------------------------------------

Ta#S12922787 111 ctcatggcaccgttgacgccggccacctctcttcaaactggggctcttgtccagatggacagtcagtgcaatgcattgggagaccgccattctgcaagtgtgtaccaaac

Ta#S13023380 111 ctcatggcaccgttgacgccggccacctctcttcaaactggggctcttgtccagatggacagtcagtgcaatgcattgggagaccgccattctgcaagtgtgtaccaaac

Ta#S12922811 108 ctcatggcaccgttgacgccggccacctctcttcaaactggggctcttgtccagatggacagtcagtgcaatgcattgggagaccgccattctgcaagtgtgtaccaaac

Ta#S13023458 108 ctcatggcaccgttgacgccggccacctctcttcaaactggggctcttgtccagatggacagtcagtgcaatgcattgggagaccgccattctgcaagtgtgtaccaaac

TAG-A --------------------------------------------------------------------------------------------------------------

Ta#S12922787 221 cttcagtttgtggatcgccagcgtactgtgtacaacatgggagctgcccgtgca**TAG**taactagagtttctagtaatatactatagcaataagagtcatgcagcatccct

Ta#S13023380 221 cttcagtttgtggatcgccagcgtactgtgtacaacatgggagctgcccgtgcatagtaactagagtttctagtaatatactatagcaataagagtcatgcagcatccct

Ta#S12922811 218 cttcagtttgtggatcgccagcgtactgtgtacaacatgggagctgcccgtgcatagtaactagagtttctagtaatatactatagcaataagagtcatgcagcatccct

Ta#S13023458 218 cttcagtttgtggatcgccagcgtactgtgtacaacatgggagctgcccgtgcatagtaactagagtttctagtaatatactatagcaataagagtcatgcagcatccct

TAG-A --------------------------------------------------------------------------------------------------------------

Ta#S12922787 331 gcatgcatcgccnganna-------aaaaaanaaa----------------------------------------------------------------aaaaaaaa---

Ta#S13023380 331 gcatgcatcgccnganna-------aaaaaanaaa----------------------------------------------------------------aaaaaaaa---

Ta#S12922811 328 gcatgcatcgccagaaaacttctgtacaacggaaagcgtgtcaactgaacttgatatatgtatgcaccattgcatgcatgttgttccgtgtagtaccagaaaataaaata

Ta#S13023458 328 gcatgcatcgccagaaaacttctgtacaacggaaagcgtgtcaactgaacttgatatatgtatgcaccattgcatgcatgttgttccgtgtagtaccagaaaataaaata

TAG-A 1 ----------------------------------------------------------------------------**CATGTTGTTCCGTGTAGTACCA**------------

Ta#S12922787 ------------------------------

Ta#S13023380 ------------------------------

Ta#S12922811 438 agggctgctactatgtatgcaggccatttg

Ta#S13023458 438 agggctgctactatgtatgcaggccatttg

TAG-A ------------------------------

**b,**

Ta#S12922787 Pro 1 mffstkmcvatimvlaltlsphgtvdaghlssnwgscpdgqsvqcigrppfckcvpnlqfvdrqrtvynmgaara

Ta#S13023380 Pro 1 mffstkmcvatimvlaltlsphgtvdaghlssnwgscpdgqsvqcigrppfckcvpnlqfvdrqrtvynmgaara

Ta#S12922811 Pro 1 mffstkmcvatimvlaltlsphgtvdaghlssnwgscpdgqsvqcigrppfckcvpnlqfvdrqrtvynmgaara

Ta#S13023458 Pro 1 mffstkmcvatimvlaltlsphgtvdaghlssnwgscpdgqsvqcigrppfckcvpnlqfvdrqrtvynmgaara

**Supplementary Fig S1: Homology between the Tag-A sequence (CATGTTGTTCCGTGTAGTACC) and the NCBI Unigene cluster Ta 2025.** a, gene sequence alignment; b, translated protein alignment. The predicted start and stop codon, and the Tag-A sequence are illustrated as all-capitals, bold and underlined.

Ta#S13023458 1 ------------------------------------aagag-----a----------t--------ccataacttaacctgaagagtg-tgacga---gataggcagc--

Ta#S16233769 1 ggtcggaattccgggtcganccacgcgtccgctacaaagac-----a----------tagagtggtccataggataacctgaagaggg-tggcgg---gctaggcagcag

Ta#S16238772 1 ccacgcgtccgca-----------------------aagac-----a----------tagaggggtccataggataacctgaagaggg-tggcgg---gctaggcagcag

Ta#S13047140 1 g-----------------------------------aggag-----acatagagtggt--------ccataggataacctgaagaggg-tggcgg---gctaggcagcag

Ta#S13235282 1 ----------------------------------------------------------------------------------cgggtg-ggacg----------------

Ta#S16240649 1 ----------------------------------------------------------------------gtctgaacttta-----t-tttcat---gcattgcatg--

Ta#S13243590 1 ------------------------------------gagtg-----g----------t--------ccataggataacctgaagaggg-tggcgg---gctaggcagcag

Ta#S13247358 1 ca----------------------------------aagac-----a----------tagagtggtccataggataacctgaagaggg-tggcgg---gctaggcagcag

Ta#S13242082 1 ata---------------------------------gagtg-----g----------t--------ccataggataacctgaagaggg-tggcgg---gctaggcagcag

Ta#S12872007 1 ---------------------------------------------------------------------------------------g-tggcgg---gctaggcagcag

Ta#S13243662 1 ccacaacggactaca---------------------aagtg-----a----------t--------ccataagataacctgaagaggg-tgacggt--gctaggcagc--

Ta#S12915914 1 agcagacata--------------------------aagtg-----a----------t--------ccataagataacctgaagaagg-tgacggtgtgctaggcagc--

Ta#S12865365 1 -----------------------------------------------------------------------------------------tgacggt--gctaggcagc--

Ta#S16235190 1 ccacgcgtccgcggac--------------------gcgtg-----g----------g--------ccataagataacctgaagaggg-tgacggt--gctaggcagc--

Ta#S16243486 1 ccacgcgtccga------------------------aagtg-----a----------t--------ccataagataacctgaagaggg-tgacggt--gctaggcagc--

Ta#S16240107 1 --------------------------------------gtg-----a----------t--------ccataagataacctgaagaggg-tgacggt--gctaggcagc--

Ta#S32690778 1 ---------------------------------------------------------------------------------------------gt---gctaggcagc--

Ta#S16228602 1 a-----------------------------------aagtg-----a----------t--------ccataagataacctgaagaggg-tgacggt--gctaggcagc--

Ta#S16237986 1 ccacgcgtccgca-----------------------aagtg-----a----------t--------ccataagataacctgaagaggg-tgacggt--gctaggcagc--

Ta#S16236956 1 cacgcgtccgcacaacggactaca------------aagtg-----a----------t--------ccataagataacctgaagaggg-tgacggt--gctaggcagc--

Ta#S16237318 1 cc----------------------------------acgcgtccgga----------t--------ccataagataacctgaagaggg-tgacggt--gctaggcagc--

Ta#S16248887 1 ccacgcgtccga------------------------aagtg-----a----------t--------ccataagataacctgaagaggg-tgacggt--gctaggcagc--

Ta#S13247442 1 ------------------------------------------------------------------------gataacctgaagaggg-tgacggt--gctaggcagc--

Ta#S13243307 1 --------------------------------------------------------------------------------gaagaggg-tgacggt--gctaggcagc--

Ta#S12993287 1 --------------------------------------------------------------------------gattcggacgaggggtgacggtgtgctaggcagc--

Ta#S12893686 1 ----------------------------------------------------------------------------------------------------taggcagc--

Ta#S13047987 1 --------------------------------------------------------------------------------tgagaggg-tgacggtgtgctaggcagc--

Ta#S12894330 --------------------------------------------------------------------------------------------------------------

Ta#S13023458 46 -cATG-ttcttctccacaaagatgtgtgttgctaccatcatggtgctagccctgacgctctcgcctcatggcaccgttgacgccggccacctctcttcaaact------g

Ta#S16233769 92 ccatg-ttcttctccacaaagatgtgtgttgctaccatcatggtgctggccctgacgctctcgcttcagggcaccgttgccgccggccgcctctttcccaactccgggtg

Ta#S16238772 69 ccagg-ttcttctccacaaagatgtgtgttgctaccatcatggtgctggccctgacgctctcgcttcagggcaccgttgccgccggccgcctctttcccaactccgggtg

Ta#S13047140 59 ccatg-ttcttctccacaaagatgtgtgttgctaccatcatggtgctggccctgacgctctcgcttcagggcaccgttgccgccggccgcctctttcccaactccgggtg

Ta#S13235282 12 --------gttgtccacccgg------------cacgtcacggaaat-gcactg----gtccgcct-----cgccattgcccgtgtctacc------aaatct------t

Ta#S16240649 30 -catg-ttgttc-----------------------------ggagc-agtactgatgct---------------------------------------------------

Ta#S13243590 48 ccatg-ttcttctccacaaagatgtgtgttgctaccatcatggtgctggccctgacgctctcgcttcagggcaccgttgccgccggccgcctctttcccaactccgggtg

Ta#S13247358 58 ccatg-ttcttctccacaaagatgtgtgttgctaccatcatggtgctggccctgacgctctcgcttcagggcaccgttgccgccggccgcctctttcccaactccgggtg

Ta#S13242082 51 ccatg-ttcttctccacaaagatgtgtgttgctaccatcatggtgctggccctgacgctctcgcttcagggcaccgttgccgccggccgcctctttcccaactccgggtg

Ta#S12872007 20 ccatg-ttcttctccacaaana-gtgtgttgctaccatcatggtgctggccctgacgctctcgcttcagggcaccgttgccgccggccgcctctttcccaactccgggtg

Ta#S13243662 62 -catg-ctattctccacagacatgtgtgttgctaccatcatggtgctggccctgacgctctcgcttcagggcaccattgacgccggccgcctctttcccaactctgggtg

Ta#S12915914 59 -catg-ttcttctccacaaagatgtgcgttgctgccatcatggtgctggccctggcgctctcgcttcagggcaccgttgacgccggccgcctctttcccaactctgggtg

Ta#S12865365 18 -catg-ctattctccacagac-tgtgtgttgctaccatc-tggtgctggccctgacgctctcgcttcagggcaccattgacgccggccgcctctttcccaactctgggtg

Ta#S16235190 63 -catg-ctattctccacagacatgtgtgttgctaccatcatggtgctggccctgacgctctcgcttcagggcaccattgacgccggccgcctctttcccaactctgggtg

Ta#S16243486 59 -catg-ctattctccacagacatgtgtgttgctaccatcatggtgctggccctgacgctctcgcttcagggcaccattgacgccggccgcctctttcccaactctgggtg

Ta#S16240107 45 -catg-ctattctccacagacatgtgtgttgctaccatcatggtgctggccctgacgctctcgcttcagggcaccattgacgccggccgcctctttcccaactctgggtg

Ta#S32690778 13 -catg-ctattctccacagacatgtgtgttgctaccatcatggtgctggccctgacgctctcgcttcagggcaccattgacgccggccgcctctttcccaactctgggtg

Ta#S16228602 48 -catg-ctattctccacagacatgtgtgttgctaccatcatggtgctggccctgacgctctcgcttcagggcaccattgacgccggccgcctctttcccaactctgggtg

Ta#S16237986 60 -catg-ctattctccacagacatgtgtgttgctaccatcatggtgctggccctgacgctctcgcttcagggcaccattgacgccggccgcctctttcccaactctgggtg

Ta#S16236956 71 -catg-ctattctccacagacatgtgtgttgctaccatcatggtgctggccctgacgctctcgcttcagggcaccattgacgccggccgcctctttcccaactctgggtg

Ta#S16237318 54 -catg-ctattctccacagacatgtgtgttgctaccatcatggtgctggccctgacgctctcgcttcagggcaccattgacgccggccgcctctttcccaactctgggtg

Ta#S16248887 59 -catg-ctattctccacagacatgtgtgttgctaccatcatggtgctggccctgacgctctcgcttcagggcaccattgacgccggccgcctctttcccaactctgggtg

Ta#S13247442 34 -catg-ctattctccacagacatgtgtgttgctaccatcatggtgctggccctgacgctctcgcttcagggcaccattgacgccggccgcctctttcccaactctgggtg

Ta#S13243307 26 -catg-ctattctccacagacatgtgtgttgctaccatcatggtgctggccctgacgctctcgcttcagggcaccattgacgccggccgcctctttcccaactctgggtg

Ta#S12993287 35 -catggttcttctccacaaagatgtgcgttgctgccatcatggtgctggccctggcgctctcgcttcagggcaccgttgacgccggccgcctctttcccaactctgggtg

Ta#S12893686 9 -catg-ttcttctccacaaagatgtgcgttgctgccatcatggtgctggccctggcgctctcgcttcagggcaccgttgacgccggccgcctctttcccaactctgggtg

Ta#S13047987 28 -catg-ttcttctccacaaagatgtgcgttgctgccatcatggtgctggccctggcgctctcgcttcagggcaccgttgacgccggccgcctctttcccaactctgggtg

Ta#S12894330 1 ---------ttctccacaaagatgtgcgttgctgccatcatggtgctggccctggcgctctcgcttcagggcaccgttgacgccggccgcctctttcccaactctgggtg

Ta#S13023458 148 gggctcttgtccagatggacag----tcagtgcaatgcattgggagaccgccattc--tgcaagtgtgtaccaaaccttcagtttgtggatcgccagcgta---------

Ta#S16233769 201 ggacggttgtccacccggcacg----tcacggaaatgcactggtccgcctcgccat--tgcccgtgtctaccaaatcttcgacttttggatcgccaagacatgatgg---

Ta#S16238772 178 ggacggttgtccacccggcacg----tcacggaaatgcactggtccgcctcgccat--tgcccgtgtctaccaaatcttcgacttttggatcgccaagacatgatgg---

Ta#S13047140 168 ggacggttgtccacccggcacg----tcacggaaatgcactggtccgcctcgccat--tgcccgtgtctaccaaatcttcgacttttggatcgccaagacatgatgg---

Ta#S13235282 80 cgacttttg----gatcgccaagacatgatggcgatgcccaagaagaacagcctcg--tgc-----tgtccc--agctatcgtgcccggaacaccaagtgt---------

Ta#S16240649 57 --gctcttgtc---------------------------------------------------------------------------------------------------

Ta#S13243590 157 ggacggttgtccacccggcacg----tcacggaaatgcactggtccgcctcgccat--tgcccgtgtctaccaaatcttcgacttttggatcgccaagacatgatgg---

Ta#S13247358 167 ggacggttgtccacccggcacg----tcacggaaatgcactggtccgcctcgccat--tgcccgtgtctaccaaatcttcgacttttggatcgcc---------------

Ta#S13242082 160 ggacggttgtccacccggcacg----tcacggaaatgcactggtccgcctcgccat--tgcccgtgtctaccaaatcttcgacttttggatcgcc---------------

Ta#S12872007 128 ggacggttgtccacccggcacg----tcacggaaatgcactggtccgcctcgccat--tgcccgtgtctaccaaatcttcgacttttggatcgcc---------------

Ta#S13243662 170 ggacggttgtccgcctggaaca----tcgcggaaatgcactggtccgcctgctcat--tgccagtgcctaccaaatcttggacctgtggatcgccaagaca---------

Ta#S12915914 167 ggacggttgtccgcctggaaca----tcgcggaaatgcactggtccgcccgctcat--tgccagtgcctaccaaatcttgggcctgtgga--------------------

Ta#S12865365 124 ggacggttgtccgcctggaaca----tcgcggaaatgcactggtccgcctgctcat--tgccagtgcctaccaaatcttggacctgtggatcgcc---------------

Ta#S16235190 171 ggacggttgtccgcctggaaca----tcgcggaaatgcactggtccgcctgctcat--tgccagtgcctaccaaatcttggacctgtggatcgccaagaca---------

Ta#S16243486 167 ggacggttgtccgcctggaaca----tcgcggaaatgcactggtccgcctgctcat--tgccagtgcctaccaaatcttggacctgtggatcgccaagaca---------

Ta#S16240107 153 ggacggttgtccgcctggaaca----tcgcggaaatgcactggtccgcctgctcat--tgccagtgcctaccaaatcttggacctgtggatcgccaagaca---------

Ta#S32690778 121 ggacggttgtccgcctggaaca----tcgcggaaatgcactggtccgcctgctcat--tgccagtgcctaccaaatcttggacctgtggatcgccaagaca---------

Ta#S16228602 156 ggacggttgtccgcctggaaca----tcgcggaaatgcactggtccgcctgctcat--tgccagtgcctaccaaatcttggacctgtggatcgccaagaca---------

Ta#S16237986 168 ggacggttgtccgcctggaaca----tcgcggaaatgcactggtccgcctgctcat--tgccagtgcctaccaaatcttggacctgtggatcgccaagaca---------

Ta#S16236956 179 ggacggttgtccgcctggaaca----tcgcggaaatgcactggtccgcctgctcat--tgccagtgcctaccaaatcttggacctgtggatcgccaagaca---------

Ta#S16237318 162 ggacggttgtccgcctggaaca----tcgcggaaatgcactggtccgcctgctcat--tgccagtgcctaccaaatcttggacctgtggatcgccaagaca---------

Ta#S16248887 167 ggacggttgtccgcctggaaca----tcgcggaaatgcactggtccgcctgctcat--tgccagtgcctaccaaatcttggacctgtggatcgccaagaca---------

Ta#S13247442 142 ggacggttgtccgcctggaaca----tcgcggaaatgcactggtccgcctgctcat--tgccagtgcctaccaaatcttggacctgtggatcgccaagaca---------

Ta#S13243307 134 ggacggttgtccgcctggaaca----tcgcggaaatgcactggtccgcctgctcat--tgccagtgcctaccaaatcttggacctgtggatcgcc---------------

Ta#S12993287 144 ggacggttgtccgcctggaaca----tcgcggaaatgcactggtccgcccgctcat--tgccagtgcctaccaaatcttgggcctgtggatcaccaagacatgacggtga

Ta#S12893686 117 ggacggttgtccgcctggaaca----tcgcggaaatgcactggtccgcccgctcat--tgccagtgcctaccaaatcttgggcctgtggatcacc---------------

Ta#S13047987 136 ggacggttgtccgcctggaaca----tcgcggaaatgcactggtccgcccgctcat--tgccagtgcctaccaaatcttgggcctgtggatcacc---------------

Ta#S12894330 102 ggacggttgtccgcctggaaca----tcgcggaaatgcactg---gtccgccgctcattgccagtgcctaccaaatcttgggcctgtggatcacca--------------

Ta#S13023458 243 ---ct------gtgtacaacatg--g--------g-agctgcccgtgc-a**TAG**taactagagtttctagta-------------------atatacta----------ta

Ta#S16233769 302 ---cg------atgcccaagaag--aacagcctcg-tgctgtcccagctatcgtgcccggaacaccaagtgtgggtgtgcgaggggggg-agatgctcctgcgagtacta

Ta#S16238772 279 ---cg------atgcccaagaag--aacagcctcg-tgctgtcccagctatcgtgcccggaacaccaagtgtgggtgtgcgaggggggg-agatgctcctgcgagtacta

Ta#S13047140 269 ---cg------atgcccaagaag--aacagcctcg-tgctgtcccagctatcgtgcccggaacaccaagtgtgggtgtgcgaggggggg-agatgctcctgcgagtacta

Ta#S13235282 168 ---gg------gtgtgcgagggg--g--------ggagatgctcctgc-----------gag-------------------------------tacta-----------a

Ta#S16240649 66 -------------------------------------------------------------gtt----------------------------------------------

Ta#S13243590 258 ---cg------atgcccaagaag--aacagcctcg-tgctgtcccagctatcgtgcccggaacaccaagtgtgggtgttgccagggggggagatgctcctgcgagtacta

Ta#S13247358 256 ---------------aagacatgatg--------g-cgatgccc-----------------------------------------------------------------a

Ta#S13242082 249 ---------------aagacatgatg--------g-cgatgccc-----------------------------------------------------------------a

Ta#S12872007 217 ---------------aagacatgatg--------g-cgatgccc-----------------------------------------------------------------a

Ta#S13243662 265 ---tgatggtggtgcccaagaag--aacagcctcg-tgctgccgcagctatcgtgcccggaacaccaagtgtgggtgtgccaggggggg-agatgctcctgcgagtacta

Ta#S12915914 --------------------------------------------------------------------------------------------------------------

Ta#S12865365 213 ---------------aagacatgatg--------g-tggtgcc-------------------------------------------------------------------

Ta#S16235190 266 ---tgatggtggtgcccaagaag--aacagcctcg-tgctgccgcagctatcgtgcccggaacaccaagtgtgggtgtgcgaggggggg-agatgctcctgcgagtacta

Ta#S16243486 262 ---tgatggtggtgcccaagaag--aacagcctcg-tgctgccgcagctatcgtgcccggaacaccaagtgtgggtgtgcgaggggggg-agatgctcctgcgagtacta

Ta#S16240107 248 ---tgatggtggtgcccaagaag--aacagcctcg-tgctgccgcagctatcgtgcccggaacaccaagtgtgggtgtgcgaggggggg-agatgctcctgcgagtacta

Ta#S32690778 216 ---tgatggtggtgcccaagaag--aacagcctcg-tgctgccccagctatcgtgcccggaacaccaagtgtgggtgtgcgaggggggggagatgctcctgcgagtacta

Ta#S16228602 251 ---tgatggtggtgcccaagaag--aacagcctcg-tgctgccgcagctatcgtgcccggaacaccaagtgtgggtgtgcgaggggggg-agatgctcctgcgagtacta

Ta#S16237986 263 ---tgatggtggtgcccaagaag--aacagcctcg-tgctgccgcagctatcgtgcccggaacaccaagtgtgggtgtgcgaggggggg-agatgctcctgcgagtacta

Ta#S16236956 274 ---tgatggtggtgcccaagaag--aacagcctcg-tgctgccgcagctatcgtgcccggaacaccaagtgtgggtgtgcgaggggggg-agatgctcctgcgagtacta

Ta#S16237318 257 ---tgatggtggtgcccaagaag--aacagcctcg-tgctgccgcagctatcgtgcccggaacaccaagtgtgggtgtgcgaggggggg-agatgctcctgcgagtacta

Ta#S16248887 262 ---tgatggtggtgcccaagaag--aacagcctcg-tgctgccgcagctatcgtgcccggaacaccaagtgtgggtgtgcgaggggggg-agatgctcctgcgagtacta

Ta#S13247442 237 ---tgatggtggtgcccaagaag--aacagcctcg-tgctgccgcagctatcgtgcccggaacaccaagtgtgggtgttgcgaggggggganatncncctgcgagtacta

Ta#S13243307 223 ---------------aagacatgatg--------g-tggtgcc-------------------------------------------------------------------

Ta#S12993287 248 tgcct------gagaagaacagc--ctc------g-tgctgccccagctatcgtgcccggaacaccaagtgtgggtgtgcgaggggggggagatgctcctgcgagtacta

Ta#S12893686 206 ---------------aagacatgacg--------g-tgatgcctgag---------------------------------------------------------------

Ta#S13047987 225 ---------------aagacatgacg--------g-tgatgcctgag---------------------------------------------------------------

Ta#S12894330 --------------------------------------------------------------------------------------------------------------

Ta#S13023458 303 gcaataagag--tcat------gcagcatccctgcatgcatcgcca-gaaaacttctgtacaacggaaagc-gtgtcaactgaacttgatat-atgtatgcaccattgca

Ta#S16233769 399 gcgataaggg--tca-------gcagcatacatccgtgcattgcca-gaaaacctctatgcacaggagagt-gtgccgtctgaactt--tat-tttcatg---cattgca

Ta#S16238772 376 gcgataaggg--tca-------gcagcatacatccgtgcattgcca-gaaaacctctatgcacaggagagt-gtgccgtctgaactt--tat-tttcatg---cattgca

Ta#S13047140 366 gcgataaggg--tca-------gcagcatacatccgtgcattgcca-gaaaacctctatgcacaggagagt-gtgccgtctgaactt--tat-tttcatg---cattgca

Ta#S13235282 206 ncgataaggg--tca-------gcagcatacatccgtgcattgcca-gaaaacctctatgcacaggagagt-gtgccgtctgaactt--tat-tttcatg---cattgca

Ta#S16240649 69 ---------g--taag------gcttcctcc-------------------------------------------------------------------tgtaccaatgaa

Ta#S13243590 356 gcgattaggg--tcaa------gcagcatacatccgtgcattgcca-gaaaacctctatgcacagganagt-gtgccgtctgaactttat------tttcaagcattgca

Ta#S13247358 277 agaagaacag--cctc------gtgctgtcccagctatcgtgcccg-gaacac-------------caagt-gtgggtgntcagggggggan-atg----------ctcc

Ta#S13242082 270 agaagaacag--cctc------gtgctgtcccanctatcgtgcccg-gaacac-------------caagt-gtg-----------------------------------

Ta#S12872007 238 agaagaacag--cctc------gtgctgtcccagctttcgtgcccg-gaacac-------------caagt-gtg-----------------------------------

Ta#S13243662 368 gcgataaggg--gca-------acagcatacatccatgcattgccaagaaaacctctatgcacaagaaagt-gtgccgtctgaactt--tat-tttcatg---cattgca

Ta#S12915914 --------------------------------------------------------------------------------------------------------------

Ta#S12865365 232 -caagaaga-------------acagcct-----cgtgctgcccca-gctatcgtgcccggaacaccaagt-gtg-----------------------------------

Ta#S16235190 369 gcgataaggg--gca-------gaagcataaatccatgcattgcca-gaaaaactctatgcacaagaaagt-gtgccgtctgaactt--tat-tttcatg---cattgaa

Ta#S16243486 365 gcgataaggg--gca-------gcagcatacatccatgcattgcca-gaaaacctctatgcacaagaaagt-gtgccgtctgaactt--tat-tttcatg---cattgca

Ta#S16240107 351 gcgataaggg--gca-------gcagcatacatccatgcattgcca-gaaaacctctatgcacaagaaagt-gtgccgtctgaactt--tat-tttcatg---cattgca

Ta#S32690778 320 gcgataaggg--gca-------gcagcatacatccatgcattgcca-gaaaacctctatgcacaagaaagt-gtgccgtctgaactt--tat-tttcatg---cattgca

Ta#S16228602 354 gcgataaggg--gca-------gcagcatacatccatgcattgcca-gaaaacctctatgcacaagaaagt-gtgccgtctgaacttcat------tttcatgcattgca

Ta#S16237986 366 gcgataaggg--gca-------gcagcatacatccatgcattgcca-gaaaacctctatgcacaagaaagt-gtgccgtctgaactt--tat-tttcatg---cattgca

Ta#S16236956 377 gcgataaggg--gca-------gcagcatacatccatgcattgcca-gaaaacctctatgcacaagaaagt-gtgccgtctgaactt--tat-tttcatg---cattgca

Ta#S16237318 360 gcgataaggg--gca-------gcagcatacatccatgcattgcca-gaaaacctctatgcacaagaaagt-gtgccgtctgaactt--tat-tttcatg---cattgca

Ta#S16248887 365 gcgataaggg--gca-------gcagcatacatccatgcattgcca-gaaaacctctatgcacaagaaagt-gtgccgtctgaactt--tat-tttcatg---cattgca

Ta#S13247442 341 ncgataaggg--ggca------gcagcatacatccatgcattgccaagaaaacctctattcacaanaaagtngtnccgtcngaacnt--tat-tttcatgca--atgcna

Ta#S13243307 242 -caagaaga-------------acagcctccgtgc--------------------------------------tgcc----------------------gcagctatccg

Ta#S12993287 343 gcgataaggg--tcagcactcagcagcatacatccgtgcgttgcca-gaagacctctatgcacaggagagt-gtgccgtctgaactttatttgatgt-----gcattgcc

Ta#S12893686 229 --aagaacagcctcgt------gctgc--cccagctatcgtgcccg-gaa--------------------------------------------------cacca-----

Ta#S13047987 248 --aagaacagcctcgt------gctgc--cccagctatcgtgcccg-gaa--------------------------------------------------cacca-----

Ta#S12894330 191 --------ag--acat------g---------------------------------------acgg--------------------tgat--------------------

Ta#S13023458 402 tgCA**-TGTTGTTCCGTGTAGT**---------------------------------------**AC**-------------------------------------**CA**gaaaataaa

Ta#S16233769 492 tgca-tgtcgttcggagcagt---------------------------------------actgatgctgctcttgtcgtcgtaaggcttcctcctgtaccaatgaataa

Ta#S16238772 469 tgca-tgttgttcggagcagt---------------------------------------actgatgctgctctggtcgttgtaaggcttcctcctgtaccaangaataa

Ta#S13047140 459 tgca-tgttgttcggagcagt---------------------------------------actgatgctgctcttgtcgttgtaaggcttcctcctgtaccaatgaataa

Ta#S13235282 299 tgca-tgttgttcggagcagt---------------------------------------actgatgctgctcttgtcgttgtaaggcttcctcctgtaccaatgaataa

Ta#S16240649 95 t-----------------------------------------------------------------------------------------------------------aa

Ta#S13243590 450 agcaatgttntttggagcagt---------------------------------------a-------------------------------------------------

Ta#S13247358 353 tgcg-agtactancgataagg---------------------------------------gt-------------------------------------cannagcatac

Ta#S13242082 322 -----gggtgttgcg-----------------------------------------------------------------------------------------------

Ta#S12872007 --------------------------------------------------------------------------------------------------------------

Ta#S13243662 462 tgca-tgctgttccg---------------------------------------------------------------------------------------------ga

Ta#S12915914 --------------------------------------------------------------------------------------------------------------

Ta#S12865365 286 ------ggtgtgc-------------------------------------------------------------------------------------------------

Ta#S16235190 462 tgca-tgctgtacgg-----------------------------------------------------------------------------------------------

Ta#S16243486 458 tgca-tgctgttcggagcagtgctgatgctgctcatgtcgttgtaaggcttcctcctgt-ac-------------------------------------cggtgaata-a

Ta#S16240107 444 tgca-tgctgtacggagcagtgctgatgctgctcatgtcgttgtaaggcttccctcctgtac-------------------------------------cggtgaata-a

Ta#S32690778 413 tgca-tgctgttcggagcagtgctgatgctgctcatgtcgttgtaaggcttcctcctgt-ac-------------------------------------cggtgaata-a

Ta#S16228602 447 tgca-tgctgttcggagcagtgctgatgctgctcatgtcgttgtaaggcttcctcctgt-ac-------------------------------------cggtgaata-a

Ta#S16237986 459 tgca-tgctgttcggagcagtgctgatgctgctcatgtcgttgtaaggcttcctcctgt-ac-------------------------------------cggtgaata-a

Ta#S16236956 470 tgca-tgctgttcggagcagtgctgatgctgctcatgtcgttgtaaggcttcctcctgt-ac-------------------------------------cggtgaata-a

Ta#S16237318 453 tgca-tgctgttcggagcagtgctgatgctgctcatgtcgttgtaaggcttcctcctgt-ac-------------------------------------cggtgaata-a

Ta#S16248887 458 tgca-tgctgttcggagcagtgctgatgctgctcatgtcgttgtaaggcttcctcctgt-ac-------------------------------------cggtgaata-a

Ta#S13247442 438 tgga-ttctgttcgg--------------------------------------------------------------------------------------gacaaatnc

Ta#S13243307 278 tgc----------------------------------------------------------c-------------------------------------cggaaca----

Ta#S12993287 444 tgca-tgctgttcggagcagt---------------------------------------ac------------------------------------------------

Ta#S12893686 273 -------------agtgtgg------------------------------------------------------------------------------------------

Ta#S13047987 292 -------------agtgt--------------------------------------------------------------------------------------------

Ta#S12894330 --------------------------------------------------------------------------------------------------------------

Ta#S13023458 435 ataagggc---tgctacta-----------tgtatgcaggccatttg-------------------------------

Ta#S16233769 562 ataagggc---cgctgcta-----------tatgcgcaggccatttgcttttagcaaaaaacc---------------

Ta#S16238772 539 ataagggc---cgctgcta-----------tatgcgcaggccatttgcttttagc-----------------------

Ta#S13047140 529 ataagggc---cgctgcta-----------tatgcgcaggccatttgcttttagcacaaaaaaaaaaaaaaaaaaaaa

Ta#S13235282 369 ataagggc---cgctgcta-----------tatgcgcaggccatttgcttttaaaaaaaggggggg------------

Ta#S16240649 98 ataagggc---cgctgcta-----------tatgcgcaggccatttgcttttagc-----------------------

Ta#S13243590 472 -------c---tgatnct----------------------nctcttgtc-----------------------------

Ta#S13247358 386 atccgtgcanttgcaagna-----------nacctctatgcacaagg-------------------------------

Ta#S13242082 332 --angggg---ggacancc-----------t---------ccttccg-------------------------------

Ta#S12872007 290 ----ggtg---tgcga-------------------gggggggagatgc------------------------------

Ta#S13243662 478 ncaattgc---tgatgc---------------tgcccangtcctttgtaangnntcccccctgtaccgg---------

Ta#S12915914 ------------------------------------------------------------------------------

Ta#S12865365 293 --gagggg---gggga--------------------------------------------------------------

Ta#S16235190 476 agaagagc----------------------ttgatgatgatcatgt--------------------------------

Ta#S16243486 528 ataagggc---cgctgcta-----------tatgcgcaggccatttgcttgaaaaaaaaaaacaaca-----------

Ta#S16240107 515 ataagggc---cgctgcta-----------tatgcgcaggcccatggctttaaaaaccacaa----------------

Ta#S32690778 483 ataanggc---cgctgcta-----------tatgcgcangccatttgcttaaaaaaaaaa------------------

Ta#S16228602 517 ataagggc---cgatgcta-----------tatgcgcaggccatttgctaaaaaaaaaacaacagggcg---------

Ta#S16237986 529 ataagggc---cgctgcta-----------tatgcgcaggccatttgct-----------------------------

Ta#S16236956 540 ataagggc---cgctgcta-----------tatgcgcaggccatttgcttg---------------------------

Ta#S16237318 523 ataagggc---cgctgcta-----------tatgcgcaggccatttgcttg---------------------------

Ta#S16248887 528 ataagggc---cgctgcta-----------tatgcgcaggccatttgctt----------------------------

Ta#S13247442 461 ntgatgcc---tgct--ca-----------tgttcnttggtaanggg-------------------------------

Ta#S13243307 289 ----------------------------------------ccaattg-------------------------------

Ta#S12993287 466 -----tga---tgctgctcatgtcggtcgttgtaaggcttcctcctgta-----------------------------

Ta#S12893686 280 -------------------------------gtgtg------------------------------------------

Ta#S13047987 ------------------------------------------------------------------------------

Ta#S12894330 206 ---------------------------------------gcc------------------------------------

**Supplementary Fig S2: Alignment of the twenty seven ESTs from the Unigene cluster Ta.40040 from the EST database at NCBI.** The sequence Ta#S13023458, one of four Unigene sequences of the Unigene cluster Ta 2025 at NCBI, was used as a reference sequence in this alignment. The predicted start and stop codon, and the Tag-A sequence (CATGTTGTTCCGTGTAGTACC) are illustrated as all-capitals, bold and underlined

**a,**

TaBaD14EST-1-M_G 1 --------gggactacacagacataaagagatccataacttaacctgaagagtgtgacgagataggcagcc**ATG**ttcttctccacaaagatgtgtgttgctaccatcatg

gn1 UG TaS129227 1 ----------------------ataaagagatccataacttaacctgaagagtgtgacgagataggcagccatgttcttctccacaaagatgtgtgttgctaccatcatg

gnl|UG|TaS129228 1 -------------------------aagagatccataacttaacctgaagagtgtgacgagataggcagccatgttcttctccacaaagatgtgtgttgctaccatcatg

gn1 UG TaS130233 1 ----------------------ataaagagatccataacttaacctgaagagtgtgacgagataggcagccatgttcttctccacaaagatgtgtgttgctaccatcatg

gn1 UG TaS130234 1 -------------------------aagagatccataacttaacctgaagagtgtgacgagataggcagccatgttcttctccacaaagatgtgtgttgctaccatcatg

TaBaD4EST-1f_C02 1 -ggaacaagggactacacagacataaagagatccataacttaacctgaagagtgtgacgagataggcagccatgttcttctccacaaagatgtgtgttgctaccatcatg

TaBaD4EST-1f_F02 1 ---gacaagggactacacagacataaagagatccataacttaacctgaagagtgtgacgagataggcagccatgttcttctccacaaagatgtgtgttgctaccatcatg

TaBaD4EST-1f_F03 1 ------------------------------------------------agagtgtgacgagataggcagccatgttcttctccacaaagatgtgtgttgctaccatcatg

TaBaD4EST-1f_G05 1 gagaacaagggactacacagacataaagagatccataacttaacctgaagagtgtgacgagataggcagccatgttcttctccacaaagatgtgtgttgctaccatcatg

TaBaD14EST-1B_E1 1 -ggaacaagggactacacagacataaagagatccataacttaacctgaagagtgtgacgagataggcagccatgttcttctccacaaagatgtgtgttgctaccatcatg

TaBaD14EST-1d_D0 1 -ggaacaagggactacacagacataaagagatccataacttawgctgaagagtgtgacgagataggcagccatgttcttctccacaaagatgtgtgttgctaccatcatg

TaBaD14EST-1d_E0 1 -ggaacaagggactacacagacataaagagatccataacttaacctgaagagtgtgacgagataggcagccatgttcttctccacaaagatgtgtgttgctaccatcatg

TaBaD14EST-1d_F0 1 -ggaacaagggactacacagacataaagagatccataacttaacctgaagagtgtgacgagataggcagccatgttcttctccacaaagatgtgtgttgctaccatcatg

TaBaD14EST-1e_G0 1 gagaacaagggactacacagacataaagagatccataacttaacctgaagagtgtgacgagataggcagccatgttcttctccacaaagatgtgtgttgctaccatcatg

TaBaD14EST1g_B06 1 -ggaacaagggactacacagacataaagagatccataacttaacctgaagagtgtgacgagataggcagccatgttcttctccacaaagatgtgtgttgctaccatcatg

TaBaD14EST-1-M_C 1 gagaacaagggactacacagacataaagagatccataacttaacctgaagagtgtgacgagatgggcagccatgttcttctccacaaagatgtgtgttgctaccatcatg

TaBaD14EST-1-M_G 103 gtgctagccctgacgctctcgcctcatggcaccgttgacgccggccacctctcttcaaactggggctcttgtccagatggacagtcagtgcaatgcattgggagaccgcc

gn1 UG TaS129227 89 gtgctagccctgacgctctcgcctcatggcaccgttgacgccggccacctctcttcaaactggggctcttgtccagatggacagtcagtgcaatgcattgggagaccgcc

gnl|UG|TaS129228 86 gtgctagccctgacgctctcgcctcatggcaccgttgacgccggccacctctcttcaaactggggctcttgtccagatggacagtcagtgcaatgcattgggagaccgcc

gn1 UG TaS130233 89 gtgctagccctgacgctctcgcctcatggcaccgttgacgccggccacctctcttcaaactggggctcttgtccagatggacagtcagtgcaatgcattgggagaccgcc

gn1 UG TaS130234 86 gtgctagccctgacgctctcgcctcatggcaccgttgacgccggccacctctcttcaaactggggctcttgtccagatggacagtcagtgcaatgcattgggagaccgcc

TaBaD4EST-1f_C02 110 gtgctagccctgacgctctcgcctcatggcaccgttgacgccggccacctctcttcaaactggggctcttgtccagatggacagtcagtgcaatgcattgggagaccgcc

TaBaD4EST-1f_F02 108 gtgctagccctgacgctctcgcctcatggcaccgttgacgccggccacctctcttcaaactggggctcttgtccagatggacagtcagtgcaatgcattgggagaccgcc

TaBaD4EST-1f_F03 63 gtgctagccctgacgctctcgcctcatggcaccgttgacgccggccacctctcttcaaactggggctcttgtccagatggacagtcagtgcaatgcattgggagaccgcc

TaBaD4EST-1f_G05 111 gtgctagccctgacgctctcgcctcatggcaccgttgacgccggccacctctcttcaaactggggctcttgtccagatggacagtcagtgcaatgcattgggagaccgcc

TaBaD14EST-1B_E1 110 gtgctagccctgacgctctcgcctcatggcaccgttgacgccggccacctctcttcaaactggggctcttgtccagatggacagtcagtgcaatgcattgggagaccgcc

TaBaD14EST-1d_D0 110 gtgctagccctgacgctctcgcctcatggcaccgttgacgccggccacctctcttcaaactggggctcttgtccagatggacagtcagtgcaatgcattgggagaccgcc

TaBaD14EST-1d_E0 110 gtgctagccctgacgctctcgcctcatggcaccgttgacgccggccacctctcttcaaactggggctcttgtccagatggacagtcagtgcaatgcattgggagaccgcc

TaBaD14EST-1d_F0 110 gtgctagccctgacgctctcgcctcatggcaccgttgacgccggccacctctcttcaaactggggctcttgtccagatggacagtcagtgcaatgcattgggagaccgcc

TaBaD14EST-1e_G0 111 gtgctagccctgacgctctcgcctcatggcaccgttgacgccggccacctctcttcaaactggggctcttgtccagatggacagtcagtgcaatgcattgggagaccgcc

TaBaD14EST1g_B06 110 gtgctagccctgacgctctcgcctcatggcaccgttgacgccggccacctctcttcaaactggggctcttgtccagatggacagtcagtgcaatgcattgggagaccgcc

TaBaD14EST-1-M_C 111 gtgctagccctgacgctctcgcctcatggcaccgttgacgccggccacctctcttcaaactggggctcttgtccagatggacagtcagtgcaatgcattgggagaccgcc

TaBaD14EST-1-M_G 213 attctgcaagtgtgtaccaaaccttcagtttgtggatcgccagcgtactgtgtacaacatgggagctgcccgtgca**TAG**taactagagtttctagtaatatactatagca

gn1 UG TaS129227 199 attctgcaagtgtgtaccaaaccttcagtttgtggatcgccagcgtactgtgtacaacatgggagctgcccgtgcatagtaactagagtttctagtaatatactatagca

gnl|UG|TaS129228 196 attctgcaagtgtgtaccaaaccttcagtttgtggatcgccagcgtactgtgtacaacatgggagctgcccgtgcatagtaactagagtttctagtaatatactatagca

gn1 UG TaS130233 199 attctgcaagtgtgtaccaaaccttcagtttgtggatcgccagcgtactgtgtacaacatgggagctgcccgtgcatagtaactagagtttctagtaatatactatagca

gn1 UG TaS130234 196 attctgcaagtgtgtaccaaaccttcagtttgtggatcgccagcgtactgtgtacaacatgggagctgcccgtgcatagtaactagagtttctagtaatatactatagca

TaBaD4EST-1f_C02 220 attctgcaagtgtgtaccaaaccttcagtttgtggatcgccagcgtactgtgtacaacatgggagctgcccgtgcatagtaactagagtttctagtaatatactatggca

TaBaD4EST-1f_F02 218 attctgcaagtgtgtaccaaaccttcagtttgtggatcgccagcgtactgtgtacaacatgggagctgcccgtgcatagtaactagagtttctagtaatatactatagca

TaBaD4EST-1f_F03 173 attctgcaagtgtgtaccaaaccttcagtttgtggatcgccagcgtactgtgtacaacatgggagctgcccgtgcatagtaactagagtttctagtaatatactatagca

TaBaD4EST-1f_G05 221 attctgcaagtgtgtaccaaaccttcagtttgtggatcgccagcgtactgtgtacaacatgggagctgcccgtgcatagtaactagagtttctagtaatatactatagca

TaBaD14EST-1B_E1 220 attctgcaagtgtgtaccaaaccttcagtttgtggatcgccagtgtactgtgtacaacatgggagctgcccgtgcatagtaactagagtttctagtaatatactatagca

TaBaD14EST-1d_D0 220 attctgcaagtgtgtaccaaaccttcagtttgtggatcgccagcgtactgtgtacaacatgggagctgcccgtgcatagtaactagagtttctagtaatatactatagca

TaBaD14EST-1d_E0 220 attctgcaagtgtgtaccaaaccttcagtttgtggatcgccagcgtactgtgtacaacatgggagctgcccgtgcatagtaactagagtttctagtaatatactatagca

TaBaD14EST-1d_F0 220 attctgcaagtgtgtaccaaaccttcagtttgtggatcgccagcgtactgtgtacaacatgggagctgcccgtgcatagtaactagagtttctagtaatatactatagca

TaBaD14EST-1e_G0 221 attctgcaagtgtgtaccaaaccttcagtttgtggatcgccagcgtactgtgtacaacatgggagctgcccgtgcatagtaactagagtttctagtaatatactatagca

TaBaD14EST1g_B06 220 attctgcaagtgtgtaccaaaccttcagtttgtggatcgccagcgtactgtgtacaacatgggaactgcccgtgcatagtaactagagtttctagtaatatactatagca

TaBaD14EST-1-M_C 221 attctgcaagtgtgtaccaaaccttcagtttgtggatcgccagcgtactgtgtacaacatgggagctgcccgtgcatagtaactagagtttctagtaatatactatagca

TaBaD14EST-1-M_G 323 ataagagtcatgcagcatccctgcatgcatcgccagaaaacttctgtacaacggaaagcgtgtcaactgaacttgatatatgtatgcaccattgcatg**CATGTTGTTCCG**

gn1 UG TaS129227 309 ataagagtcatgcagcatccctgcatgcatcgcca---------------------------------------------------------------------------

gnl|UG|TaS129228 306 ataagagtcatgcagcatccctgcatgcatcgccagaaaacttctgtacaacggaaagcgtgtcaactgaacttgatatatgtatgcaccattgcatgcatgttgttccg

gn1 UG TaS130233 309 ataagagtcatgcagcatccctgcatgcatcgcca---------------------------------------------------------------------------

gn1 UG TaS130234 306 ataagagtcatgcagcatccctgcatgcatcgccagaaaacttctgtacaacggaaagcgtgtcaactgaacttgatatatgtatgcaccattgcatgcatgttgttccg

TaBaD4EST-1f_C02 330 ataagagtcatgcagcatccctgcatgcatcgccagaaaacttctgtacaacggaaagcgtgtcaactgaacttgatatatgtatgcaccattgcatgcatgttgttccg

TaBaD4EST-1f_F02 328 ataagagtcatgcagcatccctgcatgcatcgccagaaaacttctgtacaacggaaagcgtgtcaactgaacttgatatatgtatgcaccattgcatgcatgttgttccg

TaBaD4EST-1f_F03 283 ataagagtcatgcagcatccctgcatgcatcgccagaaaacttctgtacaacggaaagcgtgtcaactgaacttgatatatgtatgcaccattgcatgcatgttgttccg

TaBaD4EST-1f_G05 331 ataagagtcatgcagcatccctgcatgcatcgccagaaaacttctgtacaacggaaagcgtgtcaactgaacttgatatatgtatgcaccattgcatgcatgttgttccg

TaBaD14EST-1B_E1 330 ataagagtcatgcagcatccctgcatgcatcgccagaaaacttctgtacaacggaaagcgtgtcaactgaacttgatatatgtatgcaccattgcatgcatgttgttccg

TaBaD14EST-1d_D0 330 ataagagtcatgcagcatccctgcatgcatcgccagaaaacttctgtacaacggaaagcgtgtcaactgaacttgatatatgtatgcaccattgcatgcatgttgttccg

TaBaD14EST-1d_E0 330 ataagagtcatgcagcatccctgcatgcatcgccagaaaacttctgtacaacggaaagcgtgtcaactgaacttgatatatgtatgcaccattgcatgcatgttgttccg

TaBaD14EST-1d_F0 330 ataagagtcatgcagcatccctgcatgcatcgccagaaaacttctgtacaacggaaagcgtgtcaactgaacttgatatatgtatgcaccattgcatgcatgttgttccg

TaBaD14EST-1e_G0 331 ataagagtcatgcagcatccctgcatgcatcgccagaaaacttctgtacaacggaaagcgtgtcaactgaacttgatatatgtatgcaccattgcatgcatgttgttccg

TaBaD14EST1g_B06 330 ataagagtcatgcagcatccctgcatgcatcgccagaaaacttctgtacaacggaaagcgtgtcaactgaacttgatatatgtatgcaccattgcatgcatgttgttccg

TaBaD14EST-1-M_C 331 ataagagtcatgcagcatccctgcatgcatcgccagaaaacttctgtacaacggaaagcgtgtcaactgaacttgatatatgtatgcaccattgcatgcatgttgttccg

TaBaD14EST-1-M_G 433 **TGTAGTACCA**gaaaataaaataagggctgctactatgtatgcaggccatttgcttttaggaaaaaaaaaaaaaaaaaaaaaaaaaaaa----------

gn1 UG TaS129227 344 ------------------------------------------------------------aaaaaaaaaaaaaaaa----------------------

gnl|UG|TaS129228 416 tgtagtaccagaaaataaaataagggctgctactatgtatgcaggccatttg----------------------------------------------

gn1 UG TaS130233 344 ------------------------------------------------------------aaaaaaaaaaaaaaaaaaaaaaaaa-------------

gn1 UG TaS130234 416 tgtagtaccagaaaataaaataagggctgctactatgtatgcaggccatttg----------------------------------------------

TaBaD4EST-1f_C02 440 tgtagtaccagaaaataaaataagggctgctactatgtatgcaggccatttgctttt--aagaaaaaaaaaaaaaaaaaaaaaaaaaaa---------

TaBaD4EST-1f_F02 438 tgtagtaccagaaaataaaataagggctgctactatgtatgcaggcc-----------------aataaa----------------------------

TaBaD4EST-1f_F03 393 tgtagtacca-------------------------------------------------caaaaaaaaaaaaaaaaaaaaaaaaaaaa----------

TaBaD4EST-1f_G05 441 tgtagtaccagaaaataaaataagggctgctactatgtatgcaggccatttgctttt----------------agcyaywgaawmaaaawatrarwra

TaBaD14EST-1B_E1 440 tgtagtaccagaaaataaaataagggctgctactatgtatgcaggccatttgcttttagcaaagaaaaaaaacaaaaaaaaaaaa-------------

TaBaD14EST-1d_D0 440 tgtagtaccagaaaataaaataagggctgctactatgtatgc--------------------------------------------------------

TaBaD14EST-1d_E0 440 tgtagtaccagaaaataaaataagggctgctactatgtatgcaggccatttgctttt------------------aaaaaraaaa-------------

TaBaD14EST-1d_F0 440 tgtagtaccagaaaataaaataagggctactactatgtatgcaggccatttgctttt--gaaaaaaaaaaaaaaaaaaaaaaaaaaaaaa--------

TaBaD14EST-1e_G0 441 tgtagtaccagaaaataaaataagggctgctactatgtatgcaggccatttgctttt----------------agcaasaa-----------------

TaBaD14EST1g_B06 440 tgtagtaccagaaaataaaataagggctgctactatgtatgcaggccatttgctttt-----aacaaaaaaaaaaaaaaaaaaaaaaa----------

TaBaD14EST-1-M_C 441 tgtagtaccagaaaataaaataagggctgctactatgtatgcaggccatttgctttta--aaaaaaaaaaaaaaaaaaaaaaaaaaaa----------

**b,**

TaBaD14EST-1-M_G 1 mffstkmcvatimvlaltlsphgtvdaghlssnwgscpdgqsvqcigrppfckcvpnlqfvdrqrtvynmgaara

gn1 UG TaS129227 1 mffstkmcvatimvlaltlsphgtvdaghlssnwgscpdgqsvqcigrppfckcvpnlqfvdrqrtvynmgaara

gn1 |UG|TaS129228 1 mffstkmcvatimvlaltlsphgtvdaghlssnwgscpdgqsvqcigrppfckcvpnlqfvdrqrtvynmgaara

gn1 UG TaS130233 1 mffstkmcvatimvlaltlsphgtvdaghlssnwgscpdgqsvqcigrppfckcvpnlqfvdrqrtvynmgaara

gn1 UG TaS130234 1 mffstkmcvatimvlaltlsphgtvdaghlssnwgscpdgqsvqcigrppfckcvpnlqfvdrqrtvynmgaara

TaBaD4EST-1f_C02 1 mffstkmcvatimvlaltlsphgtvdaghlssnwgscpdgqsvqcigrppfckcvpnlqfvdrqrtvynmgaara

TaBaD4EST-1f_F02 1 mffstkmcvatimvlaltlsphgtvdaghlssnwgscpdgqsvqcigrppfckcvpnlqfvdrqrtvynmgaara

TaBaD4EST-1f_F03 1 mffstkmcvatimvlaltlsphgtvdaghlssnwgscpdgqsvqcigrppfckcvpnlqfvdrqrtvynmgaara

TaBaD4EST-1f_G05 1 mffstkmcvatimvlaltlsphgtvdaghlssnwgscpdgqsvqcigrppfckcvpnlqfvdrqrtvynmgaara

TaBaD14EST-1B_E1 1 mffstkmcvatimvlaltlsphgtvdaghlssnwgscpdgqsvqcigrppfckcvpnlqfvdrqctvynmgaara

TaBaD14EST-1d_D0 1 mffstkmcvatimvlaltlsphgtvdaghlssnwgscpdgqsvqcigrppfckcvpnlqfvdrqrtvynmgaara

TaBaD14EST-1d_E0 1 mffstkmcvatimvlaltlsphgtvdaghlssnwgscpdgqsvqcigrppfckcvpnlqfvdrqrtvynmgaara

TaBaD14EST-1d_F0 1 mffstkmcvatimvlaltlsphgtvdaghlssnwgscpdgqsvqcigrppfckcvpnlqfvdrqrtvynmgaara

TaBaD14EST-1e_G0 1 mffstkmcvatimvlaltlsphgtvdaghlssnwgscpdgqsvqcigrppfckcvpnlqfvdrqrtvynmgaara

TaBaD14EST1g_B06 1 mffstkmcvatimvlaltlsphgtvdaghlssnwgscpdgqsvqcigrppfckcvpnlqfvdrqrtvynmgtara

TaBaD14EST-1-M_C 1 mffstkmcvatimvlaltlsphgtvdaghlssnwgscpdgqsvqcigrppfckcvpnlqfvdrqrtvynmgaara

**Supplementary Fig S3: Alignment of the sixteen ESTs generated from a 14 day post anthesis cDNA library of wheat cv Banks.**

a, gene sequence alignment; b, translated protein alignment. The predicted start and stop codon, and the Tag-A (CATGTTGTTCCGTGTAGTACC) sequence are illustrated as all-capitals, bold and underlined.

**a,**

Ta#S12922787 1 ----------------------ataaagagatccataacttaacctgaagagtgtgacgagataggcagcc**ATG**ttcttctccacaaagatgtgtgttgctaccatcatg

Ta#S13023380 1 ----------------------ataaagagatccataacttaacctgaagagtgtgacgagataggcagccatgttcttctccacaaagatgtgtgttgctaccatcatg

Ta#S12922811 1 -------------------------aagagatccataacttaacctgaagagtgtgacgagataggcagccatgttcttctccacaaagatgtgtgttgctaccatcatg

Ta#S13023458 1 -------------------------aagagatccataacttaacctgaagagtgtgacgagataggcagccatgttcttctccacaaagatgtgtgttgctaccatcatg

TaBaD14EST-1-M_G 1 --------gggactacacagacataaagagatccataacttaacctgaagagtgtgacgagataggcagccatgttcttctccacaaagatgtgtgttgctaccatcatg

gn1 UG TaS129227 1 ----------------------ataaagagatccataacttaacctgaagagtgtgacgagataggcagccatgttcttctccacaaagatgtgtgttgctaccatcatg

gnl|UG|TaS129228 1 -------------------------aagagatccataacttaacctgaagagtgtgacgagataggcagccatgttcttctccacaaagatgtgtgttgctaccatcatg

gn1 UG TaS130233 1 ----------------------ataaagagatccataacttaacctgaagagtgtgacgagataggcagccatgttcttctccacaaagatgtgtgttgctaccatcatg

gn1 UG TaS130234 1 -------------------------aagagatccataacttaacctgaagagtgtgacgagataggcagccatgttcttctccacaaagatgtgtgttgctaccatcatg

TaBaD4EST-1f_C02 1 -ggaacaagggactacacagacataaagagatccataacttaacctgaagagtgtgacgagataggcagccatgttcttctccacaaagatgtgtgttgctaccatcatg

TaBaD4EST-1f_F02 1 ---gacaagggactacacagacataaagagatccataacttaacctgaagagtgtgacgagataggcagccatgttcttctccacaaagatgtgtgttgctaccatcatg

TaBaD4EST-1f_F03 1 ------------------------------------------------agagtgtgacgagataggcagccatgttcttctccacaaagatgtgtgttgctaccatcatg

TaBaD4EST-1f_G05 1 gagaacaagggactacacagacataaagagatccataacttaacctgaagagtgtgacgagataggcagccatgttcttctccacaaagatgtgtgttgctaccatcatg

TaBaD14EST-1B_E1 1 -ggaacaagggactacacagacataaagagatccataacttaacctgaagagtgtgacgagataggcagccatgttcttctccacaaagatgtgtgttgctaccatcatg

TaBaD14EST-1d_D0 1 -ggaacaagggactacacagacataaagagatccataacttawgctgaagagtgtgacgagataggcagccatgttcttctccacaaagatgtgtgttgctaccatcatg

TaBaD14EST-1d_E0 1 -ggaacaagggactacacagacataaagagatccataacttaacctgaagagtgtgacgagataggcagccatgttcttctccacaaagatgtgtgttgctaccatcatg

TaBaD14EST-1d_F0 1 -ggaacaagggactacacagacataaagagatccataacttaacctgaagagtgtgacgagataggcagccatgttcttctccacaaagatgtgtgttgctaccatcatg

TaBaD14EST-1e_G0 1 gagaacaagggactacacagacataaagagatccataacttaacctgaagagtgtgacgagataggcagccatgttcttctccacaaagatgtgtgttgctaccatcatg

TaBaD14EST1g_B06 1 -ggaacaagggactacacagacataaagagatccataacttaacctgaagagtgtgacgagataggcagccatgttcttctccacaaagatgtgtgttgctaccatcatg

TaBaD14EST-1-M_C 1 gagaacaagggactacacagacataaagagatccataacttaacctgaagagtgtgacgagatgggcagccatgttcttctccacaaagatgtgtgttgctaccatcatg

Ta#S12922787 89 gtgctagccctgacgctctcgcctcatggcaccgttgacgccggccacctctcttcaaactggggctcttgtccagatggacagtcagtgcaatgcattgggagaccgcc

Ta#S13023380 89 gtgctagccctgacgctctcgcctcatggcaccgttgacgccggccacctctcttcaaactggggctcttgtccagatggacagtcagtgcaatgcattgggagaccgcc

Ta#S12922811 86 gtgctagccctgacgctctcgcctcatggcaccgttgacgccggccacctctcttcaaactggggctcttgtccagatggacagtcagtgcaatgcattgggagaccgcc

Ta#S13023458 86 gtgctagccctgacgctctcgcctcatggcaccgttgacgccggccacctctcttcaaactggggctcttgtccagatggacagtcagtgcaatgcattgggagaccgcc

TaBaD14EST-1-M_G 103 gtgctagccctgacgctctcgcctcatggcaccgttgacgccggccacctctcttcaaactggggctcttgtccagatggacagtcagtgcaatgcattgggagaccgcc

gn1 UG TaS129227 89 gtgctagccctgacgctctcgcctcatggcaccgttgacgccggccacctctcttcaaactggggctcttgtccagatggacagtcagtgcaatgcattgggagaccgcc

gnl|UG|TaS129228 86 gtgctagccctgacgctctcgcctcatggcaccgttgacgccggccacctctcttcaaactggggctcttgtccagatggacagtcagtgcaatgcattgggagaccgcc

gn1 UG TaS130233 89 gtgctagccctgacgctctcgcctcatggcaccgttgacgccggccacctctcttcaaactggggctcttgtccagatggacagtcagtgcaatgcattgggagaccgcc

gn1 UG TaS130234 86 gtgctagccctgacgctctcgcctcatggcaccgttgacgccggccacctctcttcaaactggggctcttgtccagatggacagtcagtgcaatgcattgggagaccgcc

TaBaD4EST-1f_C02 110 gtgctagccctgacgctctcgcctcatggcaccgttgacgccggccacctctcttcaaactggggctcttgtccagatggacagtcagtgcaatgcattgggagaccgcc

TaBaD4EST-1f_F02 108 gtgctagccctgacgctctcgcctcatggcaccgttgacgccggccacctctcttcaaactggggctcttgtccagatggacagtcagtgcaatgcattgggagaccgcc

TaBaD4EST-1f_F03 63 gtgctagccctgacgctctcgcctcatggcaccgttgacgccggccacctctcttcaaactggggctcttgtccagatggacagtcagtgcaatgcattgggagaccgcc

TaBaD4EST-1f_G05 111 gtgctagccctgacgctctcgcctcatggcaccgttgacgccggccacctctcttcaaactggggctcttgtccagatggacagtcagtgcaatgcattgggagaccgcc

TaBaD14EST-1B_E1 110 gtgctagccctgacgctctcgcctcatggcaccgttgacgccggccacctctcttcaaactggggctcttgtccagatggacagtcagtgcaatgcattgggagaccgcc

TaBaD14EST-1d_D0 110 gtgctagccctgacgctctcgcctcatggcaccgttgacgccggccacctctcttcaaactggggctcttgtccagatggacagtcagtgcaatgcattgggagaccgcc

TaBaD14EST-1d_E0 110 gtgctagccctgacgctctcgcctcatggcaccgttgacgccggccacctctcttcaaactggggctcttgtccagatggacagtcagtgcaatgcattgggagaccgcc

TaBaD14EST-1d_F0 110 gtgctagccctgacgctctcgcctcatggcaccgttgacgccggccacctctcttcaaactggggctcttgtccagatggacagtcagtgcaatgcattgggagaccgcc

TaBaD14EST-1e_G0 111 gtgctagccctgacgctctcgcctcatggcaccgttgacgccggccacctctcttcaaactggggctcttgtccagatggacagtcagtgcaatgcattgggagaccgcc

TaBaD14EST1g_B06 110 gtgctagccctgacgctctcgcctcatggcaccgttgacgccggccacctctcttcaaactggggctcttgtccagatggacagtcagtgcaatgcattgggagaccgcc

TaBaD14EST-1-M_C 111 gtgctagccctgacgctctcgcctcatggcaccgttgacgccggccacctctcttcaaactggggctcttgtccagatggacagtcagtgcaatgcattgggagaccgcc

Ta#S12922787 199 attctgcaagtgtgtaccaaaccttcagtttgtggatcgccagcgtactgtgtacaacatgggagctgcccgtgca**TAG**taactagagtttctagtaatatactatagca

Ta#S13023380 199 attctgcaagtgtgtaccaaaccttcagtttgtggatcgccagcgtactgtgtacaacatgggagctgcccgtgcatagtaactagagtttctagtaatatactatagca

Ta#S12922811 196 attctgcaagtgtgtaccaaaccttcagtttgtggatcgccagcgtactgtgtacaacatgggagctgcccgtgcatagtaactagagtttctagtaatatactatagca

Ta#S13023458 196 attctgcaagtgtgtaccaaaccttcagtttgtggatcgccagcgtactgtgtacaacatgggagctgcccgtgcatagtaactagagtttctagtaatatactatagca

TaBaD14EST-1-M_G 213 attctgcaagtgtgtaccaaaccttcagtttgtggatcgccagcgtactgtgtacaacatgggagctgcccgtgcatagtaactagagtttctagtaatatactatagca

gn1 UG TaS129227 199 attctgcaagtgtgtaccaaaccttcagtttgtggatcgccagcgtactgtgtacaacatgggagctgcccgtgcatagtaactagagtttctagtaatatactatagca

gnl|UG|TaS129228 196 attctgcaagtgtgtaccaaaccttcagtttgtggatcgccagcgtactgtgtacaacatgggagctgcccgtgcatagtaactagagtttctagtaatatactatagca

gn1 UG TaS130233 199 attctgcaagtgtgtaccaaaccttcagtttgtggatcgccagcgtactgtgtacaacatgggagctgcccgtgcatagtaactagagtttctagtaatatactatagca

gn1 UG TaS130234 196 attctgcaagtgtgtaccaaaccttcagtttgtggatcgccagcgtactgtgtacaacatgggagctgcccgtgcatagtaactagagtttctagtaatatactatagca

TaBaD4EST-1f_C02 220 attctgcaagtgtgtaccaaaccttcagtttgtggatcgccagcgtactgtgtacaacatgggagctgcccgtgcatagtaactagagtttctagtaatatactatggca

TaBaD4EST-1f_F02 218 attctgcaagtgtgtaccaaaccttcagtttgtggatcgccagcgtactgtgtacaacatgggagctgcccgtgcatagtaactagagtttctagtaatatactatagca

TaBaD4EST-1f_F03 173 attctgcaagtgtgtaccaaaccttcagtttgtggatcgccagcgtactgtgtacaacatgggagctgcccgtgcatagtaactagagtttctagtaatatactatagca

TaBaD4EST-1f_G05 221 attctgcaagtgtgtaccaaaccttcagtttgtggatcgccagcgtactgtgtacaacatgggagctgcccgtgcatagtaactagagtttctagtaatatactatagca

TaBaD14EST-1B_E1 220 attctgcaagtgtgtaccaaaccttcagtttgtggatcgccagtgtactgtgtacaacatgggagctgcccgtgcatagtaactagagtttctagtaatatactatagca

TaBaD14EST-1d_D0 220 attctgcaagtgtgtaccaaaccttcagtttgtggatcgccagcgtactgtgtacaacatgggagctgcccgtgcatagtaactagagtttctagtaatatactatagca

TaBaD14EST-1d_E0 220 attctgcaagtgtgtaccaaaccttcagtttgtggatcgccagcgtactgtgtacaacatgggagctgcccgtgcatagtaactagagtttctagtaatatactatagca

TaBaD14EST-1d_F0 220 attctgcaagtgtgtaccaaaccttcagtttgtggatcgccagcgtactgtgtacaacatgggagctgcccgtgcatagtaactagagtttctagtaatatactatagca

TaBaD14EST-1e_G0 221 attctgcaagtgtgtaccaaaccttcagtttgtggatcgccagcgtactgtgtacaacatgggagctgcccgtgcatagtaactagagtttctagtaatatactatagca

TaBaD14EST1g_B06 220 attctgcaagtgtgtaccaaaccttcagtttgtggatcgccagcgtactgtgtacaacatgggaactgcccgtgcatagtaactagagtttctagtaatatactatagca

TaBaD14EST-1-M_C 221 attctgcaagtgtgtaccaaaccttcagtttgtggatcgccagcgtactgtgtacaacatgggagctgcccgtgcatagtaactagagtttctagtaatatactatagca

Ta#S12922787 309 ataagagtcatgcagcatccctgcatgcatcgccnganna----------------------------------------------------------------------

Ta#S13023380 309 ataagagtcatgcagcatccctgcatgcatcgccnganna----------------------------------------------------------------------

Ta#S12922811 306 ataagagtcatgcagcatccctgcatgcatcgccagaaaacttctgtacaacggaaagcgtgtcaactgaacttgatatatgtatgcaccattgcatg**CATGTTGTTCCG**

Ta#S13023458 306 ataagagtcatgcagcatccctgcatgcatcgccagaaaacttctgtacaacggaaagcgtgtcaactgaacttgatatatgtatgcaccattgcatgcatgttgttccg

TaBaD14EST-1-M_G 323 ataagagtcatgcagcatccctgcatgcatcgccagaaaacttctgtacaacggaaagcgtgtcaactgaacttgatatatgtatgcaccattgcatgcatgttgttccg

gn1 UG TaS129227 309 ataagagtcatgcagcatccctgcatgcatcgcc----------------------------------------------------------------------------

gnl|UG|TaS129228 306 ataagagtcatgcagcatccctgcatgcatcgccagaaaacttctgtacaacggaaagcgtgtcaactgaacttgatatatgtatgcaccattgcatgcatgttgttccg

gn1 UG TaS130233 309 ataagagtcatgcagcatccctgcatgcatcgccaaaaaa----------------------------------------------------------------------

gn1 UG TaS130234 306 ataagagtcatgcagcatccctgcatgcatcgccagaaaacttctgtacaacggaaagcgtgtcaactgaacttgatatatgtatgcaccattgcatgcatgttgttccg

TaBaD4EST-1f_C02 330 ataagagtcatgcagcatccctgcatgcatcgccagaaaacttctgtacaacggaaagcgtgtcaactgaacttgatatatgtatgcaccattgcatgcatgttgttccg

TaBaD4EST-1f_F02 328 ataagagtcatgcagcatccctgcatgcatcgccagaaaacttctgtacaacggaaagcgtgtcaactgaacttgatatatgtatgcaccattgcatgcatgttgttccg

TaBaD4EST-1f_F03 283 ataagagtcatgcagcatccctgcatgcatcgccagaaaacttctgtacaacggaaagcgtgtcaactgaacttgatatatgtatgcaccattgcatgcatgttgttccg

TaBaD4EST-1f_G05 331 ataagagtcatgcagcatccctgcatgcatcgccagaaaacttctgtacaacggaaagcgtgtcaactgaacttgatatatgtatgcaccattgcatgcatgttgttccg

TaBaD14EST-1B_E1 330 ataagagtcatgcagcatccctgcatgcatcgccagaaaacttctgtacaacggaaagcgtgtcaactgaacttgatatatgtatgcaccattgcatgcatgttgttccg

TaBaD14EST-1d_D0 330 ataagagtcatgcagcatccctgcatgcatcgccagaaaacttctgtacaacggaaagcgtgtcaactgaacttgatatatgtatgcaccattgcatgcatgttgttccg

TaBaD14EST-1d_E0 330 ataagagtcatgcagcatccctgcatgcatcgccagaaaacttctgtacaacggaaagcgtgtcaactgaacttgatatatgtatgcaccattgcatgcatgttgttccg

TaBaD14EST-1d_F0 330 ataagagtcatgcagcatccctgcatgcatcgccagaaaacttctgtacaacggaaagcgtgtcaactgaacttgatatatgtatgcaccattgcatgcatgttgttccg

TaBaD14EST-1e_G0 331 ataagagtcatgcagcatccctgcatgcatcgccagaaaacttctgtacaacggaaagcgtgtcaactgaacttgatatatgtatgcaccattgcatgcatgttgttccg

TaBaD14EST1g_B06 330 ataagagtcatgcagcatccctgcatgcatcgccagaaaacttctgtacaacggaaagcgtgtcaactgaacttgatatatgtatgcaccattgcatgcatgttgttccg

TaBaD14EST-1-M_C 331 ataagagtcatgcagcatccctgcatgcatcgccagaaaacttctgtacaacggaaagcgtgtcaactgaacttgatatatgtatgcaccattgcatgcatgttgttccg

Ta#S12922787 349 ----------------------------------------------------------aaaaaanaaaaaaaaaaa--------------------

Ta#S13023380 349 ----------------------------------------------------------aaaaaanaaaaaaaaaaa--------------------

Ta#S12922811 416 TGTAGTACCAgaaaataaaataagggctgctactatgtatgcaggccatttg--------------------------------------------

Ta#S13023458 416 tgtagtaccagaaaataaaataagggctgctactatgtatgcaggccatttg--------------------------------------------

TaBaD14EST-1-M_G 433 tgtagtaccagaaaataaaataagggctgctactatgtatgcaggccatttgcttttaggaaaaaaaaaaaaaaaaaaaaaaaaaaaa--------

gn1 UG TaS129227 343 -----------------------------------------------------------aaaaaaaaaaaaaaaaa--------------------

gnl|UG|TaS129228 416 tgtagtaccagaaaataaaataagggctgctactatgtatgcaggccatttg--------------------------------------------

gn1 UG TaS130233 349 ----------------------------------------------------------aaaaaaaaaaaaaaaaaaaa------------------

gn1 UG TaS130234 416 tgtagtaccagaaaataaaataagggctgctactatgtatgcaggccatttg--------------------------------------------

TaBaD4EST-1f_C02 440 tgtagtaccagaaaataaaataagggctgctactatgtatgcaggccatttgcttttaagaaaaaaaaaaaaaaaaaaaaaaaaaaa---------

TaBaD4EST-1f_F02 438 tgtagtaccagaaaataaaataagggctgctactatgtatgcaggccaataaa-------------------------------------------

TaBaD4EST-1f_F03 393 tgtagtacca-------------------------------------------------caaaaaaaaaaaaaaaaaaaaaaaaaaaa--------

TaBaD4EST-1f_G05 441 tgtagtaccagaaaataaaataagggctgctactatgtatgcaggccatttgctttt--------------agcyaywgaawmaaaawatrarwra

TaBaD14EST-1B_E1 440 tgtagtaccagaaaataaaataagggctgctactatgtatgcaggccatttgcttttagcaaagaaaaaaaacaaaaaaaaaaaa-----------

TaBaD14EST-1d_D0 440 tgtagtaccagaaaataaaataagggctgctactatgtatgc------------------------------------------------------

TaBaD14EST-1d_E0 440 tgtagtaccagaaaataaaataagggctgctactatgtatgcaggccatttg---------cttttaaaaaraaaa--------------------

TaBaD14EST-1d_F0 440 tgtagtaccagaaaataaaataagggctactactatgtatgcaggccatttgcttttgaaaaaaaaaaaaaaaaaaaaaaaaaaaaaa--------

TaBaD14EST-1e_G0 441 tgtagtaccagaaaataaaataagggctgctactatgtatgcaggccatttgctttt--------------agc-----aasaa------------

TaBaD14EST1g_B06 440 tgtagtaccagaaaataaaataagggctgctactatgtatgcaggccatttgcttttaacaaaaaaaaaaaaaaaaaaaaaaa-------------

TaBaD14EST-1-M_C 441 tgtagtaccagaaaataaaataagggctgctactatgtatgcaggccatttgcttttaaaaaaaaaaaaaaaaaaaaaaaaaaaaa----------

**b,**

Ta#S12922787 1 mffstkmcvatimvlaltlsphgtvdaghlssnwgscpdgqsvqcigrppfckcvpnlqfvdrqrtvynmgaara

Ta#S13023380 1 mffstkmcvatimvlaltlsphgtvdaghlssnwgscpdgqsvqcigrppfckcvpnlqfvdrqrtvynmgaara

Ta#S12922811 1 mffstkmcvatimvlaltlsphgtvdaghlssnwgscpdgqsvqcigrppfckcvpnlqfvdrqrtvynmgaara

Ta#S13023458 1 mffstkmcvatimvlaltlsphgtvdaghlssnwgscpdgqsvqcigrppfckcvpnlqfvdrqrtvynmgaara

TaBaD14EST-1-M_G 1 mffstkmcvatimvlaltlsphgtvdaghlssnwgscpdgqsvqcigrppfckcvpnlqfvdrqrtvynmgaara

gn1 UG TaS129227 1 mffstkmcvatimvlaltlsphgtvdaghlssnwgscpdgqsvqcigrppfckcvpnlqfvdrqrtvynmgaara

gn1 UG TaS129228 1 mffstkmcvatimvlaltlsphgtvdaghlssnwgscpdgqsvqcigrppfckcvpnlqfvdrqrtvynmgaara

gn1 UG TaS130233 1 mffstkmcvatimvlaltlsphgtvdaghlssnwgscpdgqsvqcigrppfckcvpnlqfvdrqrtvynmgaara

gn1 UG TaS130234 1 mffstkmcvatimvlaltlsphgtvdaghlssnwgscpdgqsvqcigrppfckcvpnlqfvdrqrtvynmgaara

TaBaD4EST-1f_C02 1 mffstkmcvatimvlaltlsphgtvdaghlssnwgscpdgqsvqcigrppfckcvpnlqfvdrqrtvynmgaara

TaBaD4EST-1f_F02 1 mffstkmcvatimvlaltlsphgtvdaghlssnwgscpdgqsvqcigrppfckcvpnlqfvdrqrtvynmgaara

TaBaD4EST-1f_F03 1 mffstkmcvatimvlaltlsphgtvdaghlssnwgscpdgqsvqcigrppfckcvpnlqfvdrqrtvynmgaara

TaBaD4EST-1f_G05 1 mffstkmcvatimvlaltlsphgtvdaghlssnwgscpdgqsvqcigrppfckcvpnlqfvdrqrtvynmgaara

TaBaD14EST-1B_E1 1 mffstkmcvatimvlaltlsphgtvdaghlssnwgscpdgqsvqcigrppfckcvpnlqfvdrqctvynmgaara

TaBaD14EST-1d_D0 1 mffstkmcvatimvlaltlsphgtvdaghlssnwgscpdgqsvqcigrppfckcvpnlqfvdrqrtvynmgaara

TaBaD14EST-1d_E0 1 mffstkmcvatimvlaltlsphgtvdaghlssnwgscpdgqsvqcigrppfckcvpnlqfvdrqrtvynmgaara

TaBaD14EST-1d_F0 1 mffstkmcvatimvlaltlsphgtvdaghlssnwgscpdgqsvqcigrppfckcvpnlqfvdrqrtvynmgaara

TaBaD14EST-1e_G0 1 mffstkmcvatimvlaltlsphgtvdaghlssnwgscpdgqsvqcigrppfckcvpnlqfvdrqrtvynmgaara

TaBaD14EST1g_B06 1 mffstkmcvatimvlaltlsphgtvdaghlssnwgscpdgqsvqcigrppfckcvpnlqfvdrqrtvynmgtara

TaBaD14EST-1-M_C 1 mffstkmcvatimvlaltlsphgtvdaghlssnwgscpdgqsvqcigrppfckcvpnlqfvdrqrtvynmgaara

**Supplementary Fig S4:**

Alignment of the four Unigene sequences of the NCBI Unigene cluster Ta 2025 and the sixteen ESTs generated from a 14-day post anthesis cDNA library of wheat cv Banks.

a, gene sequence alignment; b, translated protein alignment. The predicted start and stop codon, and the Tag-A sequence (CATGTTGTTCCGTGTAGTACC) are illustrated as all-capitals, bold and underlined.

**Supplementary Fig S5: Agarose-gel-electrophoresis resolved Genome Walker PCR fragments and alignments of the isolated Genome Walker (GW) fragments from wheat genotypes Banks and Kite corresponding to the 5’-upstream region of the *wheat bread making* (*wbm*) gene.** Bn and Ki, Wheat genotypes Banks and Kite respectively; 1, 2, 3 and 4, GW fragments amplified using the *Eco* RV, *Dra* I, *Pvu* II and *Stu* I GW-libraries respectively is shown in (i); A, B, C and D, GW PCR fragment identified in descending order of size. The 5’-upstream sequences from each cultivar represent the longest GW sequence of a specific allele. A schematic of the homology between the GW fragments is shown in (ii) and their relatedness is shown as a dendogram in (iii). Thick bars and lines in (ii) indicate regions of high and low homology respectively. Arrow indicates 1000 bp of the DNA size marker.


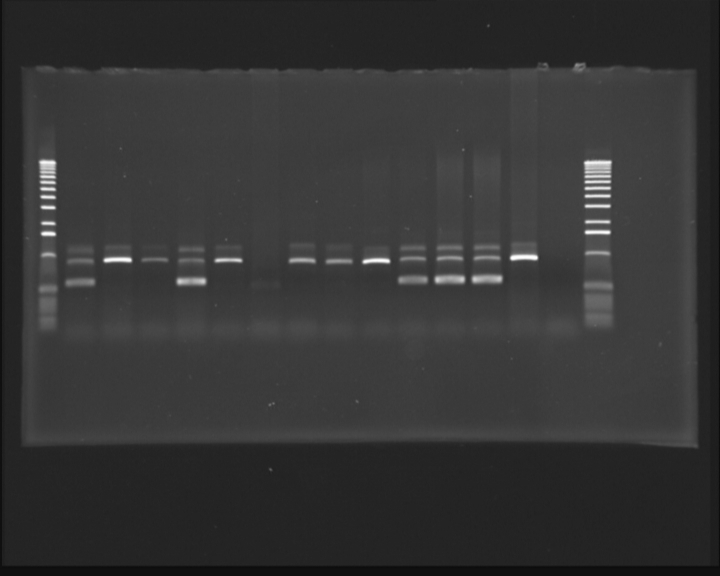


**509 bp**


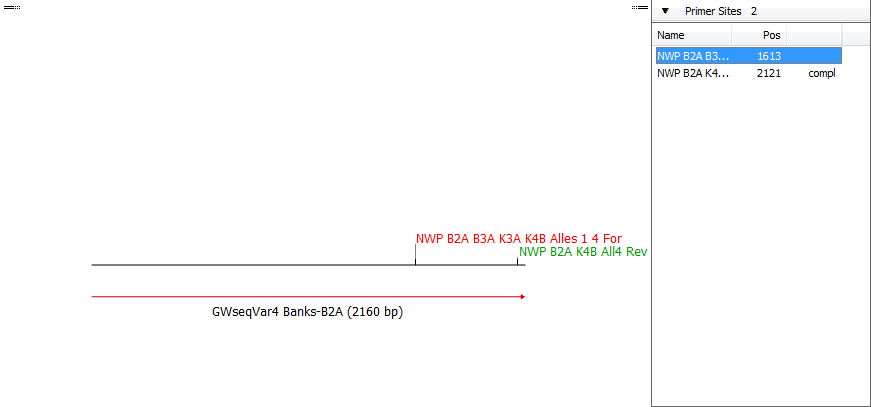

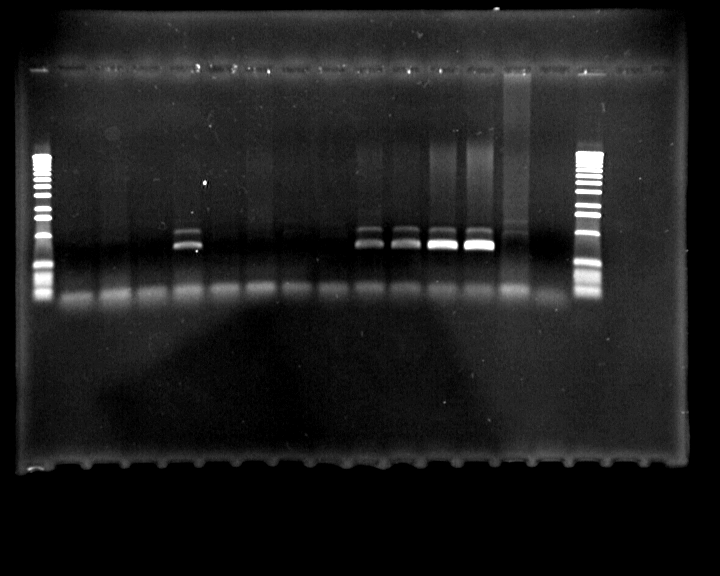


**662 bp**


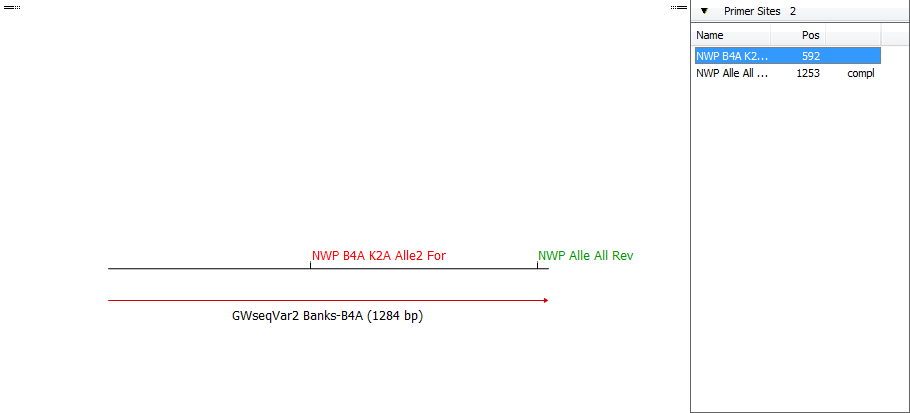

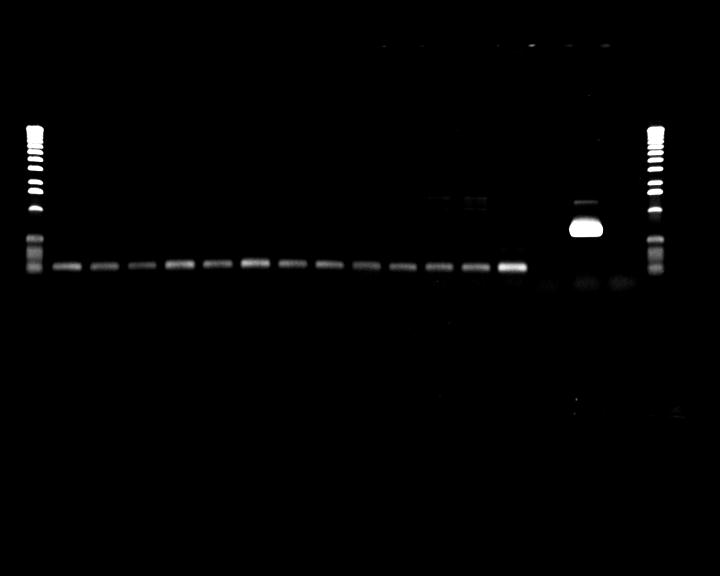


**135 bp**


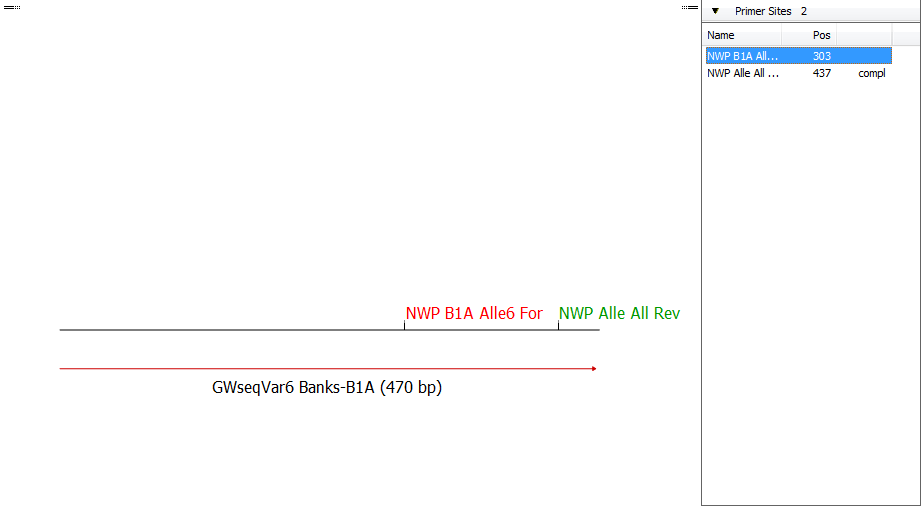


B K Bo Bl


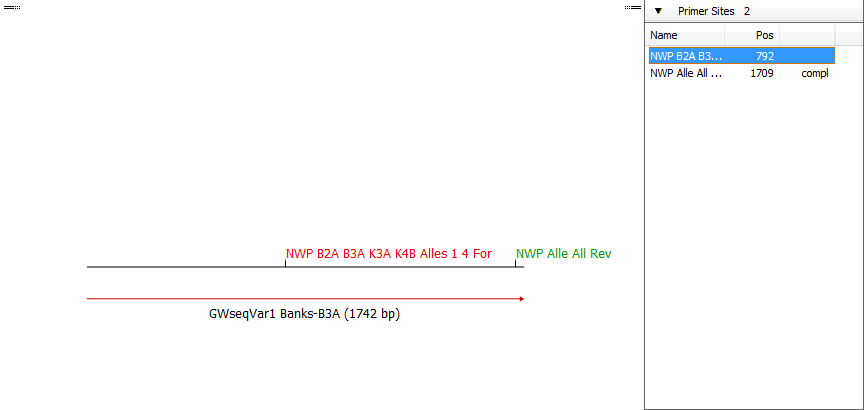


**918 bp**


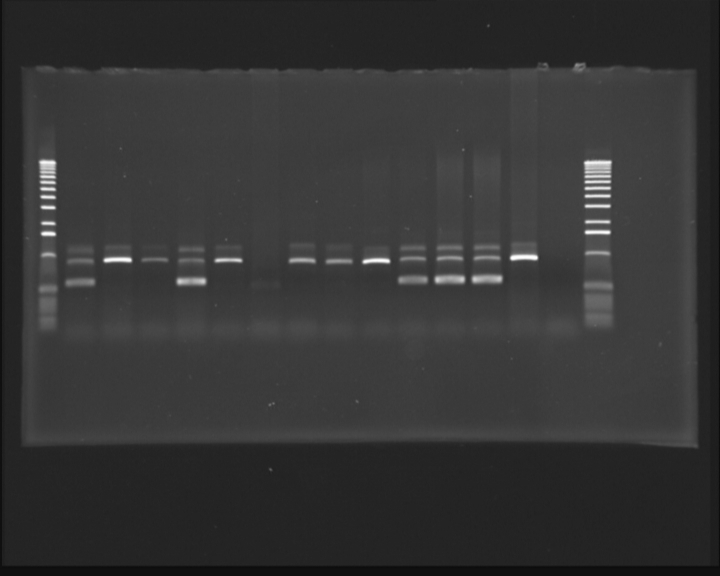

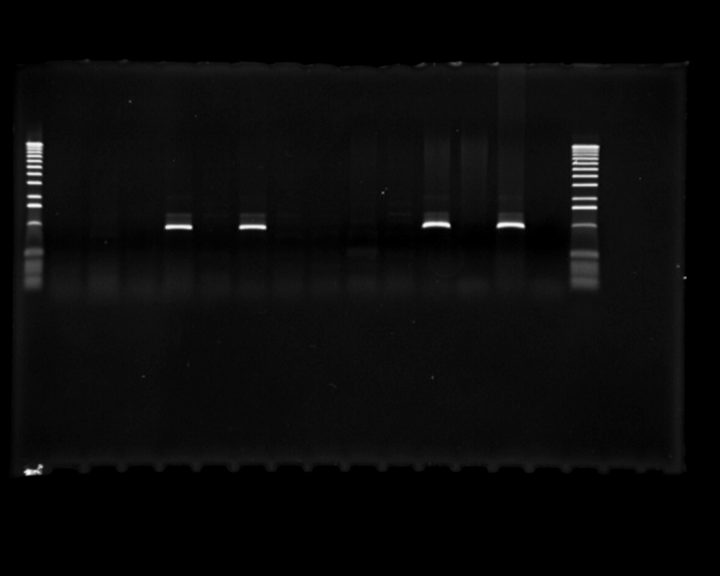


**961 bp**


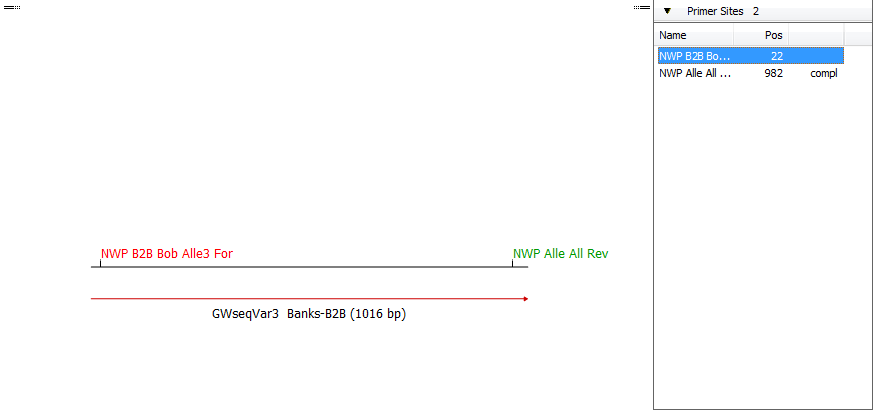


**Supplementary Fig S6:** PCR amplification of fragments of the 5’-upstream sequence variants of the *wheat bread making* (*wbm*) gene from wheat cv Banks (B), Kite (K) and Bobwhite (Bo). Bl, no template. Primers were designed to discriminate the *wmb* sequence variants based on amplified fragment sizes. A 961 bp amplified fragment of the *GWseqVar3* is present in cv Banks and Bobwhite but not in Kite.

**
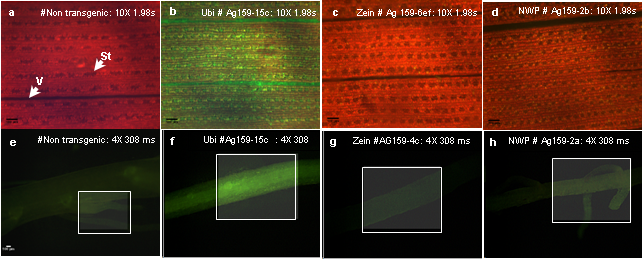
**

**Supplementary Fig S7:** Green fluorescent protein gene expression by the ubiquitin, zein and *wbm-BA3* promoters in non-seed tissues of transgenic maize plants. (a,b,c,d), leaf; (e,f,g,h), root; (a,e), non-transformed, (b,f), maize polyubiquitin ubiquitin promoter-transformed tissue; (c,g), zein promoter-transformed tissue; (d,h) *wbm-BA3* wheat promoter-transformed tissue. The *Ubi* promoter directed expression of GFP in the veins and stomata of leaf tissue and also in root tissue. The zein and the NWP-BA3 promoters do not direct GFP expression in the leaf or the root tissues. Expression of GFP was detected in all of the chlorophyll containing tissues was not conclusive due to interference from red fluorescence from chlorophyll. Detecting the expression of GFP in transgenic tissue was carried out by comparison with corresponding tissues of non-transformed plants (a,e). Observations were carried out under blue light (excitation, 489 nm; emission, 510 nm) using a compound fluorescence microscope. Red fluorescence is due to chlorophyll and yellow fluorescence is due to non-GFP-expressing living tissue and dead tissue. Representative images for promoter lines are shown and images were taken at different exposure times for clarity of images. #, line numbers of independent transgenic events; s, seconds; ms, milliseconds. Enhanced areas of images are represented as a box with a white border. Some of the composite figures may not be labelled to avoid repetition.

**
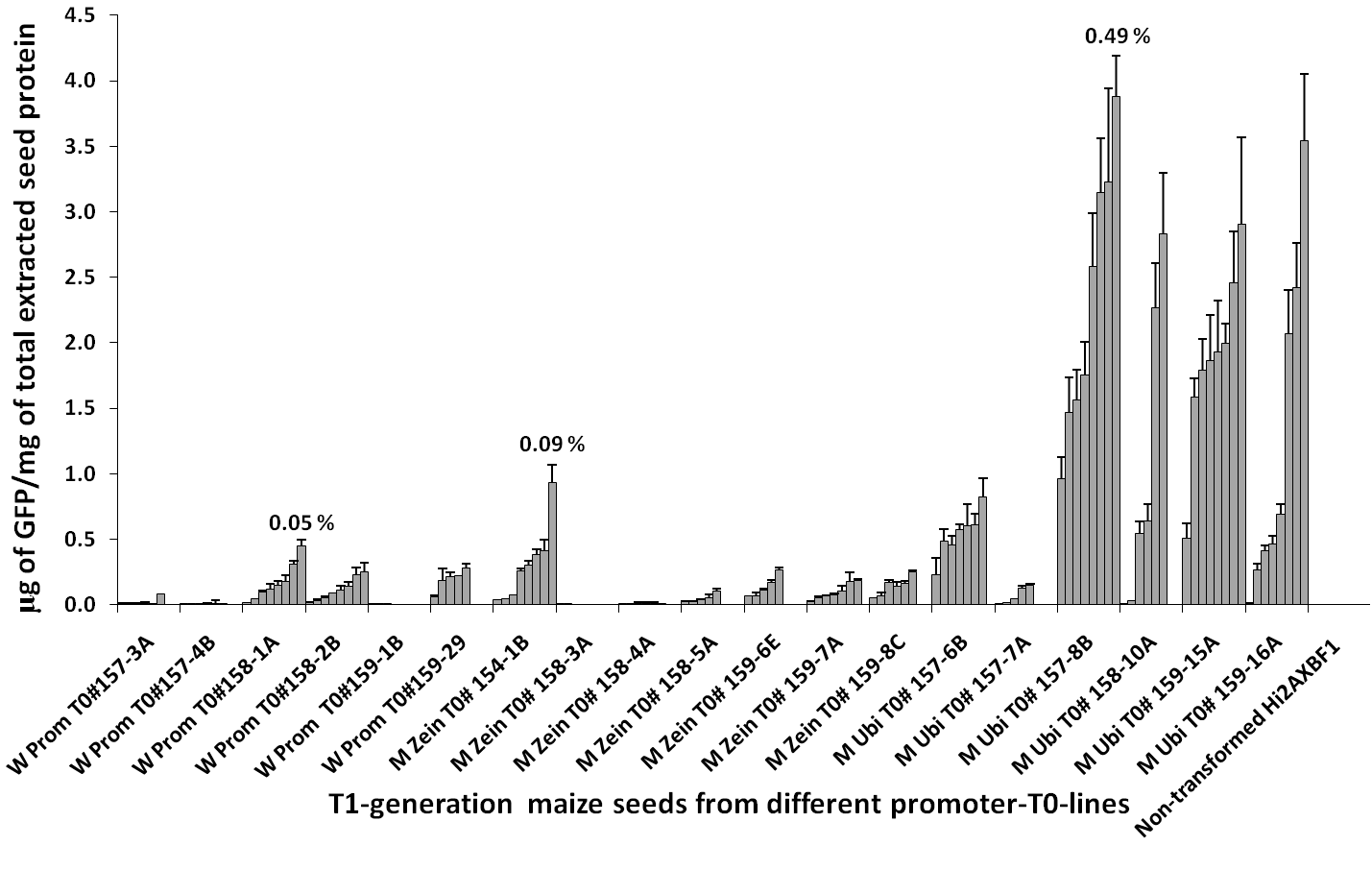
**

**Supplementary Fig S8: Measurement of expressed GFP in individual T1-generation maize seeds under control of wheat and maize promoters.** W Prom, *wbm-BA3* promoter from wheat; M Zein, *zein* promoter from maize (Accession# VO1470); M Ubi, maize polyubiquitin promoter. Measurement of GFP was carried out by ELISA in single seeds (minimum of four per transgenic line) and expressed as concentration per milligram of total extracted seed protein. Data represents values of three to six independent ELISA measurements.

**Supplementary Table S1:** High abundance tags from developing wheat seed (20, 30 days post anthesis) with the exclusion of glutenin and gliadin annotations.

| **Number** | **D20 TAG** | **Tag #** | **D30 TAG** | **Tag #** | **TAG SEQUENCE** | **Annotation** | **ID** | **ALN** | **EST?** | **HvEST** |
| --- | --- | --- | --- | --- | --- | --- | --- | --- | --- | --- |
| 1 | WBaGD20-000003 | 425 | WBaGD30-000002 | 460 | CATGTTGTTCCGTGTAGTACC | Unigene Ta.2025 Transcribed locus Triticum aestivum, 4 sequence(s) | 100 | 21 | Y |  |
| 2 | WBaGD20-000018 | 112 | WBaGD30-000014 | 136 | CATGAATAAATGTGAACTTTG | Unigene Ta.1315 Monomeric alpha-amylase inhibitor (WAI-0.28) Triticum aestivum, 204 sequence(s) | 100 | 21 | Y | S |
| 3 | WBaGD20-000016 | 116 | WBaGD30-000024 | 94 | CATGGTCTGAGATTTCACAGG | Unigene Ta.1308 Beta purothionin precursor Triticum aestivum, 138 sequence(s) | 100 | 21 | Y | M |
| 4 | WBaGD20-000021 | 102 | WBaGD30-000020 | 105 | CATGTGTGGGCTATCTGCTAT | Unigene Ta.817 Subunit CM3 of alpha-amylase tetrameric inhibitor Triticum aestivum, 426 sequence(s) | 100 | 21 | Y | M |
| 5 | WBaGD20-000019 | 106 | WBaGD30-000026 | 93 | CATGACTGAGATATAATGGTA | Unigene Ta.91 Alpha1-thionin Triticum aestivum, 143 sequence(s) | 95 | 21 | Y | M |
| 6 | WBaGD20-000032 | 70 | WBaGD30-000016 | 125 | CATGCATATATGCATATGTGA | Unigene Ta.2446 Subunit CM1 of alpha-amylase tetrameric inhibitor Triticum aestivum, 381 sequence(s) | 100 | 21 | Y | S |
| 7 | WBaGD20-000039 | 66 | WBaGD30-000022 | 101 | CATGGGCGTTGCGCCTTTTTC | UniGene Ta.154 Transcribed locus, moderately similar to XP_469153.1 putative globulin (with alternative splicing) [Oryza sativa (japonica cultivar-group)] | 100 | 21 | N | S |
| 8 | WBaGD20-000024 | 89 | WBaGD30-000029 | 78 | CATGTGCCTGCGCGAGGTGTC | Unigene Ta.23141 Puroindoline-a (pTa43) Triticum aestivum, 471 sequence(s) | 100 | 21 | Y | S |
| 9 | WBaGD20-000054 | 42 | WBaGD30-000019 | 108 | CATGGGCACTCCCAGTGTTTA | Unigene Ta.9227 Type 1 non-specific lipid transfer protein precursor (ltp9.1b gene) Triticum aestivum, 1489 sequence(s) | 100 | 21 | N | M |
| 10 | WBaGD20-000027 | 77 | WBaGD30-000033 | 73 | CATGTGTGGTGCTATGTGCTA | Unigene Ta.58 Chloroform/methanol-soluble (CM16) protein Triticum aestivum, 270 sequence(s) | 100 | 21 | Y | M |
| 11 | WBaGD20-000025 | 87 | WBaGD30-000042 | 56 | CATGGGTGGGGAGCTCCCTGC | Unigene Ta.27780 Beta amylase Triticum aestivum, 541 sequence(s) | 100 | 21 | Y | M |
| 12 | WBaGD20-000030 | 74 | WBaGD30-000036 | 65 | CATGTTGCTTCTTGGCTGCTG | Unigene Ta.28327 Transcribed locus, weakly similar to NP_922231.1 putative lipid transfer protein [Oryza sativa (japonica cultivar-group)] Triticum aestivum, 104 sequence(s) | 100 | 21 | N | M |
| 13 | WBaGD20-000061 | 36 | WBaGD30-000027 | 93 | CATGTGTGGCCAGTACTATTG | Unigene Ta.905 Transcribed locus, weakly similar to XP_468609.1 putative ribosomal protein L27a [Oryza sativa (japonica cultivar-group)] Triticum aestivum, 341 sequence(s) | 100 | 21 | N | M |
| 14 | WBaGD20-000031 | 73 | WBaGD30-000043 | 55 | CATGGGCGCCGACTGCAAGTT | Unigene Ta.115 Puroindoline-b Triticum aestivum, 170 sequence(s) | 100 | 21 | Y | S |
| 15 | WBaGD20-000037 | 67 | WBaGD30-000040 | 57 | CATGGGTGGGGAGCTCCCTCC | Unigene Ta.27780 Beta amylase Triticum aestivum, 541 sequence(s) | 100 | 21 | Y | S |
| 16 | WBaGD20-000047 | 51 | WBaGD30-000037 | 64 | CATGGAATAATATATACTTTA | Unigene Ta.69 GSP-1a mRNA for grain softness protein Triticum aestivum, 228 sequence(s) | 100 | 21 | Y | S |
| 17 | WBaGD20-000036 | 67 | WBaGD30-000047 | 46 | CATGCACCTACCTCTTAATTT | Ta.23141 Alpha-amylase inhibitor 0.19 (0.19 gene), cultivated variety Wyuna Triticum aestivum, 314 sequence(s) | 100 | 21 | Y | M |
| 18 | WBaGD20-000038 | 67 | WBaGD30-000052 | 41 | CATGTTATCCATACAATAAAT | Unigene Ta.1314 Serpin WZS2 Triticum aestivum, 190 sequence(s) | 100 | 21 | Y | S |
| 19 | WBaGD20-000046 | 52 | WBaGD30-000048 | 46 | CATGGTGGTGACGGGTGACGG | >gi|472511|gb|M82356.1|WHTRRE03 Triticum aestivum 18S ribosomal RNA (18S rRNA) bp 315 to 543 in mature rRNA | 100 | 21 | Y |  |
| 20 | WBaGD20-000100 | 17 | WBaGD30-000030 | 78 | CATGTGTGGCAAGTACTAGTT | Unigene Ta.27777 Seed storage protein Triticum aestivum, 191 sequence(s) | 100 | 21 | N | M |
| 21 | WBaGD20-000041 | 60 | WBaGD30-000059 | 34 | CATGGCCGTTCTTAGTTGGTG | Unigene Ta.384 Transcribed locus, moderately similar to XP_468456.1 putative NAC2 [Oryza sativa (japonica cultivar-group)] Triticum aestivum, 36 sequence(s) | 100 | 21 | N |  |
| 22 | WBaGD20-000042 | 57 | WBaGD30-000060 | 33 | CATGCACCTGCCTCTTAATTT | Ta.23141 Alpha-amylase inhibitor 0.19 (0.19 gene), cultivated variety Wyuna Triticum aestivum, 314 sequence(s) | 100 | 21 | N | S |
| 23 | WBaGD20-000069 | 31 | WBaGD30-000041 | 56 | CATGGAATGAAATAAAGTGGA | Unigene Ta.9226 Pathogenesis-related protein 4 (PR4) Triticum aestivum, 179 sequence(s) | 100 | 21 | N | M |
| 24 | WBaGD20-000044 | 53 | WBaGD30-000062 | 33 | CATGGTAAGTGAATGGATAAT | Unigene Ta.1304 WSCI proteinase inhibitor Triticum aestivum, 41 sequence(s) | 100 | 21 | Y | M |
| 25 | WBaGD20-000045 | 53 | WBaGD30-000064 | 32 | CATGTTACCGATACAATAAAT | Unigene Ta.1314 Serpin WZS2 Triticum aestivum, 190 sequence(s) | 100 | 21 | Y | S |
| 26 | WBaGD20-000043 | 53 | WBaGD30-000068 | 29 | CATGCGGAAATGACAGTGATA | Unigene Ta.9402 19 kDa globulin Triticum aestivum, 160 sequence(s) | 100 | 21 | Y | M |
| 27 | WBaGD20-000050 | 49 | WBaGD30-000066 | 31 | CATGTGTGGTGCTATCTGCTA | Unigene Ta.23798 CM 17 protein Triticum aestivum, 128 sequence(s) | 100 | 21 | Y | S |
| 28 | WBaGD20-000058 | 39 | WBaGD30-000058 | 35 | CATGTTAACCATACAATAAAT | Unigene Ta.118 Serpin (WSZ1c gene) Triticum aestivum, 25 sequence(s) | 100 | 21 | Y | S |
| 29 | WBaGD20-000055 | 42 | WBaGD30-000063 | 32 | CATGGGCGGGGAGCTCCTTGC | probably Ta.27780 Beta amylase Triticum aestivum, 543 sequence(s) | 100 | 21 | N | S |
| 30 | WBaGD20-000096 | 18 | WBaGD30-000044 | 53 | CATGTGTGGCCAGTACCGTTG | Unigene Ta.28312 Triticin precursor Triticum aestivum, 465 sequence(s) | 100 | 21 | N | S |
| 31 | WBaGD20-000057 | 41 | WBaGD30-000067 | 30 | CATGCCCCGATGAGTAGGAGG | Unigene Hv.12681 RNA for trypsin inhibitor Cme Hordeum vulgare, 612 sequence(s) | 100 | 21 | Y |  |
| 32 | WBaGD20-000060 | 36 | WBaGD30-000061 | 33 | CATGCATATATATATGAACAA | Unigene Ta.2446 Subunit CM1 of alpha-amylase tetrameric inhibitor Triticum aestivum, 381 sequence(s) | 100 | 21 | Y | S |
| 33 | WBaGD20-000053 | 42 | WBaGD30-000073 | 26 | CATGGGATAACATCATAGGAT | >emb|AJ272181.1|TAE272181 Triticum aestivum 18S rRNA gene for 18S ribosomal RNA strain MV-15 Length = 1764 | 100 | 21 | Y |  |
| 34 | WBaGD20-000083 | 23 | WBaGD30-000050 | 43 | CATGGAAATAATGAACAGATG | probably Ta.2448 Hypothetical LOC542894 Triticum aestivum, 143 sequence(s) | 100 | 21 | N | S |
| 35 | WBaGD20-000067 | 31 | WBaGD30-000056 | 35 | CATGGAATAATATGTACTTTA | Unigene Ta.28607 GSP-1c mRNA for grain softness protein Triticum aestivum, 75 sequence(s) | 100 | 21 | N | M |
| 36 | WBaGD20-000051 | 47 | WBaGD30-000095 | 17 | CATGTAAAACCTTGCGTTGCT | Unigene Ta.242 Small subunit ADP glucose pyrophosphorylase Triticum aestivum, 362 sequence(s) | 100 | 21 | Y | M |
| 37 | WBaGD20-000104 | 16 | WBaGD30-000049 | 44 | CATGTGTGGCAAATACTAGTT | Unigene Ta.27777 Seed storage protein Triticum aestivum, 191 sequence(s) | 100 | 21 | N | S |
| 38 | WBaGD20-000059 | 37 | WBaGD30-000077 | 23 | CATGTTATAACAAAATCTCAG | Unigene Ta.5839 Transcribed locus, weakly similar to XP_466439.1 putative glycine-rich protein [Oryza sativa (japonica cultivar-group)] Triticum aestivum, 26 sequence(s) | 100 | 21 | N | S |
| 39 | WBaGD20-000062 | 35 | WBaGD30-000082 | 21 | CATGACTGCCTACAATGGTGG | Unigene Ta.6175 Transcribed locus Triticum aestivum, 16 sequence(s) | 100 | 21 | N |  |
| 40 | WBaGD20-000093 | 18 | WBaGD30-000054 | 37 | CATGCATATGCACGACTGTGC | UniGene Ta.25053 Triticum aestivum Thaumatin-like protein | 100 | 21 | N |  |
| 41 | WBaGD20-000111 | 13 | WBaGD30-000053 | 40 | CATGGAACGAAATAAAGTGGA | Unigene Ta.9226 Pathogenesis-related protein 4 (PR4) Triticum aestivum, 179 sequence(s) | 100 | 21 | N | M |
| 42 | WBaGD20-000079 | 27 | WBaGD30-000072 | 26 | CATGCCCAACATAAATAAACA | Unigene Ta.154 Transcribed locus, moderately similar to XP_469153.1 putative globulin (with alternative splicing) [Oryza sativa (japonica cultivar-group)] Triticum aestivum, 513 sequence(s) | 100 | 21 | N | M |
| 43 | WBaGD20-000075 | 29 | WBaGD30-000083 | 21 | CATGCATCTGCCTCTTAATTT | Ta.23141 Alpha-amylase inhibitor 0.19 (0.19 gene), cultivated variety Wyuna Triticum aestivum, 314 sequence(s) | 100 | 21 | Y | S |
| 44 | WBaGD20-000063 | 34 | WBaGD30-000104 | 15 | CATGAATAATGCCGCGCGCTT | Unigene Ta.13439 Transcribed locus, weakly similar to NP_910046.1 putative Bowman-Birk serine protease inhibitor [Oryza sativa (japonica cultivar-group)] Triticum aestivum, 22 sequence(s) | 100 | 21 | Y | S |
| 45 | WBaGD20-000064 | 34 | WBaGD30-000108 | 15 | CATGGACTAAAATAAACACAT | Unigene Ta.28312 Triticin precursor Triticum aestivum, 465 sequence(s) | 100 | 21 | Y | M |
| 46 | WBaGD20-000074 | 29 | WBaGD30-000085 | 20 | CATGAGAATAAAAGACAAAGA | >gi|63252970|emb|AJ937839.2| Triticum aestivum partial mRNA for putative omega-gliadin (gli gene) | 100 | 21 | Y | S |
| 47 | WBaGD20-000090 | 19 | WBaGD30-000070 | 27 | CATGCCACGCGTTGCTTTTGG | Unigene Ta.18720 Clone wem1c.pk001.g11:fis, full insert mRNA sequence Triticum aestivum, 436 sequence(s) | 100 | 21 | N | M |
| 48 | WBaGD20-000081 | 27 | WBaGD30-000092 | 18 | CATGTGATGTTTGATGGCTTG | Unigene Ta.28304 Transcribed locus, moderately similar to XP_477421.1 putative B12D protein [Oryza sativa (japonica cultivar-group)] Triticum aestivum, 340 sequence(s) | 100 | 21 | N | M |
| 49 | WBaGD20-000078 | 28 | WBaGD30-000110 | 15 | CATGTCGAAATAATAAAAGTG | Unigene Ta.10140 Peroxidase 1 (WSP1) Triticum aestivum, 56 sequence(s) | 100 | 21 | N | M |
| 50 | WBaGD20-000082 | 24 | WBaGD30-000087 | 19 | CATGTCACAGAGTGACTGAAT | Ta.13191 Transcribed locus, weakly similar to NP_909413.1 putative protein synthesis inhibitor II (Ribosome-inactivating protein II) (rRNA N-glycosidase) [Oryza sativa (japonica cultivar-group)] Triticum aestivum, 33 sequence(s) | 100 | 21 | N | M |
| 51 | WBaGD20-000065 | 32 | WBaGD30-000147 | 10 | CATGCAATAAATCCAATAAAT | Unigene Ta.117 Serpin WZS3 Triticum aestivum, 73 sequence(s) | 100 | 21 | N | S |
| 52 | WBaGD20-000077 | 28 | WBaGD30-000114 | 13 | CATGGCGTGTCGGAGGGACAG | Unigene Ta.23141 Puroindoline-a (pTa43) Triticum aestivum, 471 sequence(s) | 100 | 21 | Y | M |
| 53 | WBaGD20-000094 | 18 | WBaGD30-000084 | 21 | CATGTAGGCGGCAACAATGGC | Unigene Ta.1480 Transcribed locus Triticum aestivum, 138 sequence(s) | 100 | 21 | N | S |
| 54 | WBaGD20-000091 | 19 | WBaGD30-000086 | 20 | CATGTGCGGAAAATATGGCAA | Unigene Ta.28318 Itrl-2 mRNA for CMx Triticum aestivum, 74 sequence(s) | 100 | 21 | Y | M |
| 55 | WBaGD20-000076 | 28 | WBaGD30-000144 | 10 | CATGATCCAATACCAGGACTC | Unigene Ta.5839 Transcribed locus, weakly similar to XP_466439.1 putative glycine-rich protein [Oryza sativa (japonica cultivar-group)] Triticum aestivum, 26 sequence(s) | 100 | 21 | Y | S |
| 56 | WBaGD20-000084 | 23 | WBaGD30-000107 | 15 | CATGGAATAATATATACTATA | Unigene Ta.840 GSP-1b mRNA for grain softness protein Triticum aestivum, 101 sequence(s) | 100 | 21 | N | M |
| 57 | WBaGD20-000102 | 16 | WBaGD30-000078 | 22 | CATGTAGGAGGCAACAACAGC | Ta.1480 Transcribed locus Triticum aestivum, 138 sequence(s) | 100 | 21 | N | S |
| 58 | WBaGD20-000071 | 30 | WBaGD30-000298 | 5 | CATGCATAAGTGCGCAATAAA | Unigene Ta.31022 WCI proteinase inhibitor Triticum aestivum, 61 sequence(s) | 100 | 21 | N | M |
| 59 | WBaGD20-000087 | 21 | WBaGD30-000113 | 14 | CATGTTGTTGTAAGGCTACCT | Unigene Ta.1272 Transcribed locus Triticum aestivum, 23 sequence(s) | 100 | 21 | N | S |
| 60 | WBaGD20-000085 | 23 | WBaGD30-000137 | 11 | CATGTCGGTCGTTGTAAGGCT | Unigene Ta.22978 Transcribed locus Triticum aestivum, 13 sequence(s) | 100 | 21 | Y | S |
| 61 | WBaGD20-000101 | 16 | WBaGD30-000093 | 17 | CATGCCACGCGTTGCTTCCAG | Unigene Ta.18720 Clone wem1c.pk001.g11:fis, full insert mRNA sequence Triticum aestivum, 436 sequence(s) | 100 | 21 | N | M |

Tags are in descending order of total abundance. The “EST?” column indicates whether a locally generated, full length EST exists that matches the tag sequence. The “HvEST” column indicates a single (S) or multiple (M) match to a HvEST gene cluster consensus sequence.
